# Genome-wide signatures of differential DNA methylation in pediatric acute lymphoblastic leukemia

**DOI:** 10.1186/gb-2013-14-9-r105

**Published:** 2013-09-24

**Authors:** Jessica Nordlund, Christofer L Bäcklin, Per Wahlberg, Stephan Busche, Eva C Berglund, Maija-Leena Eloranta, Trond Flaegstad, Erik Forestier, Britt-Marie Frost, Arja Harila-Saari, Mats Heyman, Ólafur G Jónsson, Rolf Larsson, Josefine Palle, Lars Rönnblom, Kjeld Schmiegelow, Daniel Sinnett, Stefan Söderhäll, Tomi Pastinen, Mats G Gustafsson, Gudmar Lönnerholm, Ann-Christine Syvänen

**Affiliations:** 1 Department of Medical Sciences, Molecular Medicine and Science for Life Laboratory, Uppsala University, Uppsala 75185, Sweden; 2 Department of Medical Sciences, Cancer Pharmacology and Computational Medicine, Uppsala University, Uppsala 75185, Sweden; 3 Department of Human Genetics, McGill University, Montréal, Québec H3A0G1, Canada; 4 Department of Medical Sciences, Rheumatology, Uppsala University, Uppsala 75185, Sweden; 5 Department of Pediatrics, Tromsø University and University Hospital, Tromsø N-9038, Norway; 6 Department of Medical Biosciences, University of Umeå, Umeå 90185, Sweden; 7 Department of Women’s and Children’s Health, Pediatric Oncology, Uppsala University, Uppsala 75185, Sweden; 8 Department of Pediatrics and Adolescence, Oulu University Hospital, Oulu 90029, Finland; 9 Childhood Cancer Research Unit, Karolinska Institutet, Astrid Lindgren Children’s Hospital, Karolinska University Hospital, Stockholm 17176, Sweden; 10 Pediatric Hematology-Oncology, Children’s Hospital, Barnaspitali Hringsins, Landspitali University Hospital, Reykjavik 101, Iceland; 11 ediatrics and Adolescent Medicine, Rigshospitalet, and the Medical Faculty, Institute of Clinical Medicine, University of Copenhagen, Copenhagen 2100, Denmark; 12 Division of Hematology-Oncology, CHU Sainte-Justine Research Center, Department of Pediatrics, University of Montreal, Montréal, Québec, Canada; 13 Department of Human Genetics, McGill University and Genome Quebec Innovation Center, Montréal, Québec H3T1C5, Canada; 14 For the Nordic Society of Pediatric Hematology and Oncology (NOPHO

## Abstract

**Background:**

Although aberrant DNA methylation has been observed previously in acute lymphoblastic leukemia (ALL), the patterns of differential methylation have not been comprehensively determined in all subtypes of ALL on a genome-wide scale. The relationship between DNA methylation, cytogenetic background, drug resistance and relapse in ALL is poorly understood.

**Results:**

We surveyed the DNA methylation levels of 435,941 CpG sites in samples from 764 children at diagnosis of ALL and from 27 children at relapse. This survey uncovered four characteristic methylation signatures. First, compared with control blood cells, the methylomes of ALL cells shared 9,406 predominantly hypermethylated CpG sites, independent of cytogenetic background. Second, each cytogenetic subtype of ALL displayed a unique set of hyper- and hypomethylated CpG sites. The CpG sites that constituted these two signatures differed in their functional genomic enrichment to regions with marks of active or repressed chromatin. Third, we identified subtype-specific differential methylation in promoter and enhancer regions that were strongly correlated with gene expression. Fourth, a set of 6,612 CpG sites was predominantly hypermethylated in ALL cells at relapse, compared with matched samples at diagnosis. Analysis of relapse-free survival identified CpG sites with subtype-specific differential methylation that divided the patients into different risk groups, depending on their methylation status.

**Conclusions:**

Our results suggest an important biological role for DNA methylation in the differences between ALL subtypes and in their clinical outcome after treatment.

## Background

Methylation of cytosine (5 mC) residues in CpG dinucleotides across the genome is an epigenetic modification that plays a pivotal role in the establishment of cellular identity by influencing gene expression during development [1]. In somatic mammalian cells, the majority of CpG sites are methylated. However, CpG sites located in regions of increased CG density, known as CpG islands, generally have low levels of CpG methylation [2]. On the molecular level, it is well known that CpG methylation leads to X-chromosome inactivation, genomic imprinting, and suppression of transposable elements. Disruption of DNA methylation patterns is associated with diseases, and particularly with cancer [3]. Key regulators that are essential for establishing and maintaining the epigenomic landscape are frequently mutated and can drive cancer development via alterations of DNA methylation and histone modifications [4].

Pediatric acute lymphoblastic leukemia (ALL) originates from the malignant transformation of lymphocyte progenitor cells into leukemic cells in the B-cell and T-cell lineages. ALL is a heterogeneous disease, in which patients are stratified into subtype groups based on their cellular immunophenotype and recurrent cytogenetic aberrations, such as aneuploidies and translocations, acquired by the leukemic cells [5,6]. In the Nordic countries, the five-year survival rate for pediatric ALL patients exceeds 80%, but one-fifth of the patients relapse despite continued chemotherapy [5]. Although the cytogenetic aberrations are indicative of better or poorer relapse-free survival rates, relapses occur in all cytogenetic subtypes [6].

We and others have previously observed differential patterns of CpG site methylation in ALL cells compared to non-leukemic bone marrow [7,8], in subtypes of ALL [9-12], and between diagnosis and relapse [13]. However, the genome-wide DNA methylation patterns have not yet been comprehensively described for all subtypes of ALL and the synergy between DNA methylation, leukemogenesis, drug resistance, and relapse in ALL is poorly understood. Increased understanding of the role of aberrant DNA methylation is of considerable interest, especially in lieu of the possible application of epigenetic treatment in combination chemotherapy [14,15]. In the present study we provide a comprehensive, genome-wide map of *de novo* DNA methylation changes in ALL cells at diagnosis and relapse by interrogating the methylation levels of 435,941 CpG sites distributed genome-wide in a large collection of pediatric ALL cells of diverse cytogenetic backgrounds.

## Results

### The DNA methylation landscape in ALL

HumanMethylation 450k BeadChips were used for quantitative DNA methylation analysis of leukemic blasts from pediatric ALL patients in the Nordic countries. This large collection includes samples from patients with T-cell ALL (T-ALL; n = 101) and B-cell precursor ALL (BCP ALL; n = 663), including multiple samples from rare subtypes of BCP ALL (Table 1). To determine signatures of differential methylation that are characteristic for ALL, we compared the CpG site methylation levels in ALL cells to those in blood cells from non-leukemic individuals. To represent the different stages in lymphoid cell development, we included CD19+ B cells, CD3+ T cells, and CD34+ hematopoietic stem cells isolated from healthy adult blood donors. We also included age-matched bone marrow (BM) samples collected at remission from 86 of the ALL patients as control samples. This set of non-leukemic reference cells includes multipotent progenitor cells (CD34+) and mature lymphoid cells (CD19+, CD3+), which allows the distinction of lineage- and cell type-specific differences from *de novo* methylation in the ALL cells.

**Table 1:** Clinical information for the acute lymphoblastic leukemia patients included in the study

**Clinical feature**^**a**^	**BCP ALL (%)**	**T-ALL (%)**
Number of patients	663	101
Male:female ratio	1.2	2.9
Median age (years)	4.8	9.4
High hyperdiploid (HeH)^b^	187 (30%)	3 (3%)
t(12;21)*ETV6/RUNX1*^c^	163 (26%)	0 (0%)
Undefined^d^	105 (17%)	54 (54%)
Non-recurrent^e^	100 (16%)	37 (37%)
11q23/*MLL*^c^	28 (4.5%)	4 (4%)
t(1;19)*TCF3/PBX1*^c^	23 (3.5%)	0 (0%)
dic(9;20)^c^	20 (3%)	0 (0%)
t(9;22)*BCR/ABL1*	19 (3%)	1 (<1%)
iAMP21^c^	10 (1.5%)	0 (0%)
<45 chromosomes	5 (<1%)	0 (0%)
>67 chromosomes	3 (<1%)	2 (2%)
First relapse^f^	24	3
Second relapse^f^	5	0

^a^The diagnosis was established at a pediatric oncology center by analysis of bone marrow aspirates with respect to morphology, immunophenotype, and cytogenetics of the leukemic cells. Immunophenotypes (BCP ALL or T-ALL) were defined according to the European Group for the Immunological Characterization of Leukemias. Chromosome banding of bone marrow and/or peripheral blood samples was performed using standard methods. The definition and description of clonal abnormalities followed the recommendations of International System for Human Cytogenetic Nomenclature. Karyotypes were centrally reviewed.

^b^High hyperdiploidy (HeH) was defined as a modal number more than 50 chromosomes.

^c^Fluorescence *in situ* hybridization and/or reverse-transcriptase polymerase chain reaction were applied to identify t(12;21), t(1;19), 11q23, dic(9;20)(p11-13;q11), and iAMP(21q22).

^d^Undefined includes patients with no karyotype information available.

^e^Non-recurrent includes patients with chromosomal abnormalities other than those defined in the recurrent groups.

^f^Detailed information on relapse samples is available in Additional file 2: Table S15.

**Figure 1:**
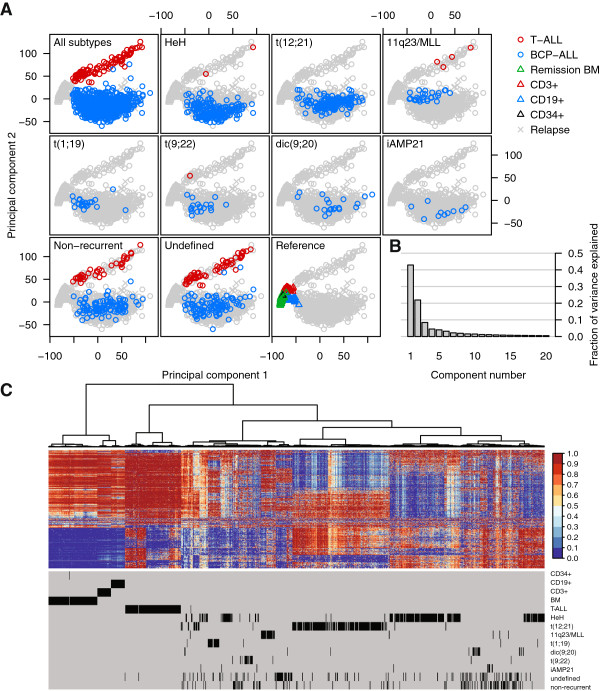
**Unsupervised analysis of DNA methylation in acute lymphoblastic leukemia (ALL) samples and non-leukemic reference samples. (A)** Principal component analysis (PCA) of the DNA methylation data for 435,941 CpG sites across all samples included in the study. The data from 764 ALL patients and 137 reference samples are plotted using the first two principal components. The top left panel shows the data for the ALL samples, with each individual sample indicated by a ring. Data from BCP ALL samples are shown in blue and data from T-ALL samples are in red. In each panel, the data from the samples with the indicated cytogenetic subtype of ALL are highlighted. The data from the four different cell types in the reference cell panel are plotted by triangles with the cell types indicated by the color key to the right of the panels. **(B)** The fraction of the variance explained by each principal component. The two first PCs shown in **(A)** explain approximately 63% of the variance in methylation levels. **(C)** Hierarchical clustering of the ALL and reference samples based on the methylation levels of 435,941 CpG sites. The 1,000 most variable CpG sites are shown in the heatmap. Clustering of samples by cell type and cytogenetic profiles is shown below the heatmap.

To obtain an initial view of the variation in CpG site methylation in our dataset, we subjected the complete set of methylation data to principal component analysis (PCA). T-ALL, BCP ALL, and non-leukemic samples formed separated clusters using the principal components 1 and 2 (Figure 1A). Although only two components were needed to capture >60% of the variation in the dataset (Figure 1B), higher order components separated the subtypes of BCP ALL from each other (not shown). Although the non-leukemic reference samples originated from different blood cell populations, they clustered together, clearly separated from the ALL samples. Unsupervised cluster analysis across all of the CpG sites revealed distinct methylation patterns that separated ALL cells according to their cytogenetic and immunophenotypic subtype. The evident difference between ALL cells and the non-leukemic blood cells, and the similarity between the non-leukemic cells in the heatmap (Figure 1C) provide the rationale to use these cells as a non-leukemic reference cell panel to detect differential methylation.

### Differential DNA methylation

**Figure 2:**
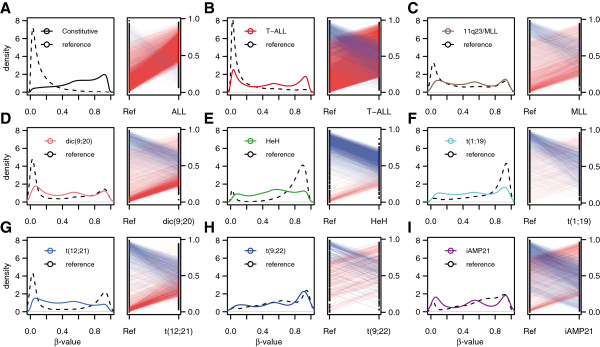
**Beta-value distribution and magnitude of differential methylation.** Density plots demonstrate the differences in β-value distribution of the differentially methylated CpGs (DMCs) in the different DMC signatures between the ALL patients and reference samples. In each panel, the distribution of all DMC β-values across all patients and controls is plotted on the left side of each panel and the mean difference in β-value for each CpG is plotted on the right side of the panel. The red lines indicate DMCs with increased DNA methylation in ALL and blue lines indicate DMCs with decreased DNA methylation in the line plots. The scales on the x-axis of the density plots and the y-axis of the line plots range from 0 (no methylation) to 1.0 (100% methylation). **(A)** The distribution of all β-values in the constitutive DMC signature for the 137 reference samples (black dashed line) and 774 ALL samples (black solid line) (left) and the mean difference in β-values (right). **(B-I)** The β-value distributions of each of the DMCs in the subtype-specific DMC signatures across the reference samples (n = 137, dashed lines) and the ALL samples by immunophenotypic or cytogenetic subtype (colored lines, left). The mean difference in methylation between the reference panel and each subtype is plotted to the right.

We searched for differentially methylated CpG sites (DMCs) in the ALL cells by comparing the β-values (methylation values ranging from 0.0 to 1.0) in non-leukemic reference samples to the ALL samples of each individual subtype. CD19+, CD34+, and BM samples were used as the reference panel for BCP ALL and CD3+, CD34+, and BM were used as the reference panel for T-ALL. For calling a CpG site as differentially methylated, we required a minimum absolute ∆β-value of 0.2 and a false discovery rate (FDR)-adjusted Wilcoxon rank-sum *P*-value of <0.01 for the difference. This analysis revealed between 21,799 and 58,157 DMCs in the ALL subtypes, distributed across 5,956 to 8,245 gene regions (Table 2; in Additional file 1: Table S1). In total, 9,406 of the DMCs annotated to 2,023 gene regions and 2,979 CpG islands were observed across all the ALL subtypes and were thereby considered 'constitutive' (Additional file 2: Table S2). The vast majority of the constitutive DMCs (98.6%) were hypermethylated in the ALL cells compared with the non-leukemic reference cells (Figure 2A). The number of DMCs that were unique for each ALL subtype according to the applied criteria varied independently of the number of samples in a subtype, from 16,841 CpG sites in 895 unique gene regions in T-ALL to 271 CpG sites in 36 unique gene regions in the t(9;22) subtype (Table 2). As expected, the heterogeneous BCP ALL samples with unknown cytogenetic aberrations labeled as 'undefined' and those with 'non-recurrent' abnormalities did not display unique differential methylation patterns. The methylation patterns between BCP ALL subtypes differed substantially, with high methylation levels in samples harboring *MLL* rearrangements, which is opposite to a recent finding of predominant hypomethylation in adult ALL with *MLL* rearrangements [9], while the high hyperdiploid (HeH) samples were predominantly hypomethylated in our study (Table 2; Figure 2B-I), as has been previously described in pediatric BCP ALL for HeH [11]. The distribution between hyper- and hypomethylation between the subtypes of pediatric BCP ALL in our study is in agreement with the findings in a recent study of 50,000 CpG sites that used an alternative method for DNA methylation analysis [16]. For the DMCs, the absolute average β-value difference between ALL cells and reference cells for the subtype-specific DMCs was approximately 0.50, which is in agreement with allele-specific gains or losses of DNA methylation in ALL compared to normal cells (Figures 2A-I; Additional file 3: Figures S1A-F).

**Table 2:** Differentially methylated CpG sites in the cytogenetic subtypes of ALL

**DMC signature**	**DMCs**^**a**^	**Genes**^**b**^	**Genes unique**	**DMCs unique**	**Unique DMCs **	**Unique DMCs**
**(number of patients)**			**to subtype**^**c**^	**to subtype**	**+ DNAm (%)**^**d**^	**-DNAm (%)**^**e**^
Constitutive (774)	9,406	2,023	NA	NA	NA	NA
T-ALL (101)	58,157	8,245	895	16,841	15,487 (92.0)	1,365 (8.0)
*MLL*/11q23 (28)	31,403	7,142	300	1,763	1,285 (72.9)	478 (27.1)
dic(9;20) (20)	53,680	9,009	202	2,370	1,561 (65.9)	809 (34.1)
HeH (187)	42,779	7,773	271	3,014	268 (8.9)	2,746 (91.1)
t(1;19)*TCF3/PBX1*(23)	21,799	5,956	107	1,110	272 (24.5)	838 (75.5)
t(12;21)*ETV6/RUNX1*(163)	45,589	7,973	156	2,114	1,126 (53.3)	988 (46.7)
t(9;22)*BCR/ABL1*(19)	23,871	6,047	36	271	140 (51.7)	131 (48.3)
iAMP21 (10)	44,726	8,614	272	2,656	997 (37.5)	1,659 (62.5)
Undefined (105)	39,262	7,059	3	56	8 (14.3)	48 (85.7)
Non-recurrent (100)	42,109	7,434	2	27	14 (51.9)	13 (48.1)

^a^Differentially methylated CpG sites (DMCs) with mean Δβ-values >0.20 and false discovery rate-corrected Wilcoxon rank-sum *P*-values <0.01.

^b^The number of gene regions to which the DMCs are annotated.

^c^Genes with DMCs are considered unique to a subtype if only that subtype had significant DMCs in the gene region.

^d^Hypermethylated DMCs, + DNA methylation (DNAm).

^e^Hypomethylated DMCs, - DNA methylation (DNAm).

### Functional genomic distribution of differentially methylated CpG sites

**Figure 3:**
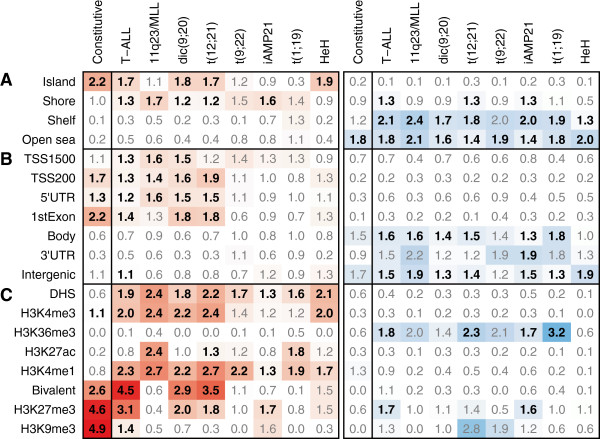
**Functional genomic annotation of differentially methylated CpGs (DMCs).** This figure shows the enrichment of the different DMC signatures. The columns show the levels of enrichment of the constitutive DMC signature shared by all subtypes of ALL and the subtype-specific DMC signatures as indicated above the panel. Functional genomic regions of DMCs annotated **(A)** in relation to gene region, **(B)** in relation to CpG island annotation, and **(C)** in relation to chromatin marks in reference cell types. The fold enrichment of each annotation is indicated in each box. The color scale in the panels indicates fold enrichment of the hypermethylated (red) or hypomethylated (blue) DMCs in each functionally annotated region. The bolded numbers indicate annotations to which DMCs are enriched compared to the distribution of probes on the 450k array (Bonferroni corrected one-sided Fisher’s exact *P* < 0.001).

The hypermethylated DMCs were enriched in CpG islands, while hypomethylated DMCs were primarily annotated to 'open sea' regions, independent of whether they were constitutive or subtype-specific (Figure 3A). The subtype-specific differences were more frequently observed in CpG island 'shores' and 'shelves', which display a large variation in β-value between ALL samples (Additional file 3: Figure S2). Both constitutive and subtype-specific DMCs in proximal promoter regions (transcription start sites and 5’ untranslated regions) of genes were commonly hypermethylated, but a greater enrichment of subtype-specific hypomethylation was observed in gene bodies and in intergenic regions (Figure 3B). To explore putative functional roles for the DMCs, we intersected the genomic coordinates of the constitutive and subtype-specific DMCs with regions defined by chromatin-immunoprecipitation of six histone marks and DNase1 hypersensitivity (DHS) assays in relevant primary cell types such as CD19+, CD3+, and CD34+ cells [17,18]. Although the histone code in normal blood cells may not reflect that in ALL cells, the genomic distribution of histone marks is useful for annotating functional regions of the genome. This analysis revealed differences in enrichment between constitutive and subtype-specific DMCs to functional genomic regions with marks of repressed or active chromatin (Figure 3C). The 9,406 constitutive DMCs were enriched more than two-fold in regions marked by repressive H3K9me3 and H3K27me3, or bivalently by H3K27me3 and H3K4me3, which marks active chromatin (*P* < 0.001). On the contrary, the subtype-specific DMCs were enriched more than two-fold in regions of active chromatin marked by DHS, H3K4me3, and H3K4me1 (*P* < 0.001; Figure 3C). These observations suggest that subtype-specific methylation of CpG sites has specific functional roles.

The constitutive DMCs were enriched in genes in the transcriptional regulatory network in embryonic stem cells (*P* = 3.53 × 10^-3^) and in genes that regulate or are regulated by transcription factors involved in embryonic development: NANOG (*P* = 9.7 × 10^-6^), OCT4 (*P* = 4.9 × 10^-5^), SOX2 (*P* = 2.3 × 10^-6^), and REST (*P* = 4.75 × 10^-13^) (Additional file 2: Table S3). While no enrichment to known pathways was observed for the subtype-specific DMC signatures, all of the DMC signatures were enriched for genes with biological functions in cancer, cellular development, cellular growth and proliferation, and cell-to-cell signaling (*P* < 0.05).

### DMCs as regulators of gene expression

To investigate whether the DMCs influence gene expression and to determine which of the annotation classes of DMCs are involved in the regulation of gene expression, we compared the DNA methylation levels of each constitutive and subtype-specific DMC with gene expression data. First, we determined the correlation between the methylation levels of constitutive DMCs and mRNA expression levels obtained using digital gene expression sequencing of 28 ALL samples, including T-ALL and five BCP ALL subtypes, and five reference samples [19] (Additional file 2: Table S4). The β-values of only a small proportion (<1%) of the constitutive DMCs (n = 85) correlated with up- or down-regulation of the mRNA expression levels of 41 genes (permuted *P* ≤ 0.05 and fold change ≥2) (Additional file 2: Table S5). This observation was expected since 79% of the constitutive DMCs were annotated to regions containing the repressive H3K27me3 or H3K9me3 marks in healthy blood cells and thus genes in these regions were presumably not widely expressed (Figure 3C). Secondly, we determined which of the subtype-specific DMCs correlated with microarray-based gene expression data for 93 of the ALL samples of the t(12;21), HeH, t(1;19), t(9;22), dic(9;20), *MLL*/11q23 and T-ALL subtypes (Additional file 2: Table S6). We found that, on average, 15% (range 10 to 21%) of the β-values for the subtype-specific DMCs annotated to genes correlated with gene expression levels (permuted *P* ≤ 0.05 and fold change ≥2) (Additional file 2: Tables S7 to S13). The proportion of DMCs and gene annotations in t(12;21) that were correlated with gene expression in our study were highly similar to those in a recent, small methylation study on the t(12;21) BCP ALL subtype [12]. Ten of the 17 genes suggested in the earlier study based on their correlation to be drivers of leukemogenesis were also highlighted in our study (Additional file 2: Table S14).

**Figure 4:**
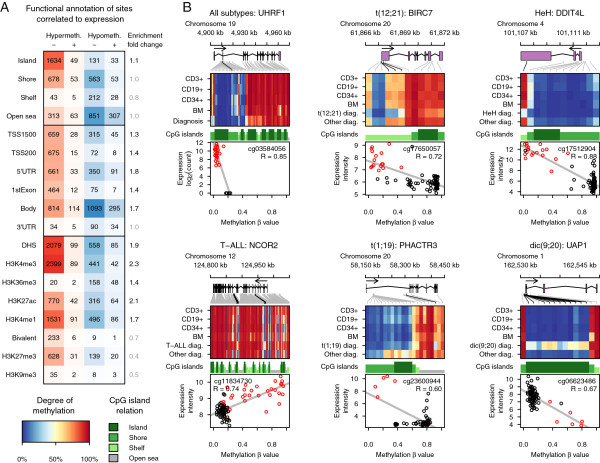
**Correlation between differentially methylated CpGs (DMCs) and gene expression. (A)** The functional genomic annotation of DMCs that are correlated with gene expression. The columns show hypermethylated DMCs in red and hypomethylated DMCs in blue, with the number of DMCs correlated with up-regulation (+) or down-regulation (-) of gene expression indicated by the numbers in the panel. The rows show the genomic annotation of the DMCs in relation to gene region, CpG island annotation, and chromatin marks in reference cell types. The fold enrichment of the DMCs within each annotation class is shown to the right of each row. The bolded numbers indicate enrichment compared to the distribution of probes on the 450k array (Bonferroni corrected one-sided Fisher’s exact *P* < 0.001). **(B)** Examples of genes with a strong correlation between their expression levels and the β-values of constitutive DMCs (upper left panel) and subtype-specific DMCs (all other panels). The ALL subgroup highlighted and the gene name and structure are outlined above each panel. The positions of the CpG sites correlated with gene expression (permuted *P* < 0.05) are indicated by black lines connecting the gene structure to the heatmap below. For each gene, the mean β-value of the probes is plotted in the heatmap for the non-leukemic cell types and ALL subtypes as indicated to the left of the heatmap. At the bottom of each panel, the mRNA expression levels are plotted on the y-axis and β-values for the highest ranking CpG site in each gene are plotted on the x-axis. Each individual sample of the subtype analyzed is indicated by red circles and other subtypes are indicated by black circles. The correlation coefficient and CpG ID are shown in each panel. The color keys for the methylation heatmap and CpG island annotation are shown at the bottom left-hand corner of the figure.

We used the functional annotation of the DMCs correlated with gene expression to explore their putative functional roles, and found hypermethylated DMCs that correlated with down-regulation of gene expression to be enriched in DHS regions, active promoters (H3K4me3), and enhancers (H3K27ac/H3K4me1) (Figure 4A; Additional file 3: Figure S3). On the contrary, hypomethylation of gene bodies was highly correlated with either up- or down-regulation of gene expression. DMCs that were highly correlated with gene expression included genes with functions in epigenetic regulation and previously known subtype-specific gene expression in ALL (Figure 4B). For example, we observed an inverse correlation between the β-value and gene expression for the *UHRF1* gene, which encodes a methyl CpG binding protein that has high affinity for hemi-methylated DNA and was highly expressed in the ALL samples, independent of their subtype, while it was not expressed in reference samples [20]. DNA methylation of *NCOR2*, which is a transcriptional co-repressor that acts through covalent modification of histones [21], was positively correlated with gene expression in T-ALL. We also show up-regulation of known subtype-specific genes such as *BIRC7* in t(12;21) [12,22] and *DDIT4L* in HeH [19], and previously unobserved subtype-specific expression of *PHACTR3* in t(1;19) and *UAP1* in the dic(9;20) subtype.

### Differential DNA methylation in relapsed ALL

**Figure 5:**
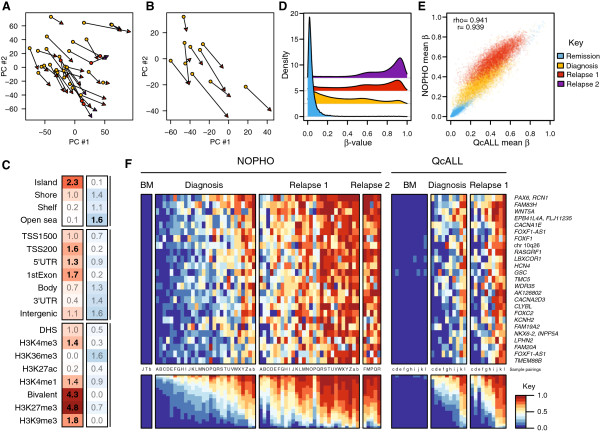
**Increased DNA methylation at relapse. (A)** Plot of the first two principal components (PC) of the genome-wide DNA methylation data in 27 diagnostic-first/second relapse paired samples. The diagnostic sample is indicated by a filled yellow circle and the last relapse sample is indicated by an arrowhead. **(B)** PCA of the 10 diagnostic-first relapse paired samples in the Quebec childhood ALL (QcALL) dataset. **(C)** Enrichment of DMCs in relation to gene region, CpG island annotation, and chromatin marks. Hypermethylated DMCs are shown in red and hypomethylated DMCs are shown in blue. In each box the fold enrichment for each specific mark is shown. Bold numbers indicate annotations enriched for relapse DMCs compared to the distribution of probes on the 450k array (Bonferroni corrected one sided Fisher’s exact *P* < 0.001). **(D)** Distribution of the methylation β-values in the relapse signature at remission (n = 3), diagnosis (n = 27), first relapse (n = 27), and second relapse (n = 5). **(E)** The mean β-values of the DMCs in the relapse signature in the Nordic Society of Pediatric Hematology and Oncology (NOPHO) and QcALL datasets. The color legend for **(A,B,D,E)** is to the right of **(E)**. **(F)** Heatmap of the top ranked relapse DMCs. From the 27 patients in the NOPHO dataset, paired remission BM was available from 3, diagnostic samples were available from 27, first relapse data were available from 27, and second relapse data were available from 5 individuals. From the QcALL dataset, DNA samples from remission BM, diagnosis and relapse were available from each of the 10 patients. Each DMC is plotted as an individual row. Each column represents one DNA sample. The proportion of methylated CpG sites is shown in the bottom panel. The gene annotations of the CpG sites are given on the right vertical axis. The color key for the methylation levels is provided at the bottom right.

Next we compared the genome-wide DNA methylation levels between paired samples at diagnosis and relapse from 27 patients, and in five of the patients after a second relapse (Additional file 2: Table S15). We used PCA to visualize the genome-wide methylation patterns of the sample pairs. Plots of the first two principal components showed similar changes in DNA methylation levels between diagnosis, first, and second relapse in all patients (Figure 5A; Additional file 3: Figure S4). We observed a similar pattern in 10 paired BCP ALL samples at diagnosis and relapse from the Quebec childhood ALL (QcALL) cohort that were included for verification of our results (Figure 5B).

In total, we identified 6,612 DMCs in 1,854 gene regions in the 27 paired diagnosis-relapse ALL samples (Additional file 2: Table S16). Although only 773 (12%) DMCs at relapse overlapped with the constitutive DMCs, the gene region annotations of both signatures were remarkably similar, and included 1,186 (64%) of overlapping gene regions. Hence, like the genes in the constitutive signature, the genes in the relapse signature were enriched for the transcriptional regulatory network in embryonic stem cells and in the Wnt/β-catenin signaling pathways (*P* = 2.8 × 10^-7^, 1.8 × 10^-4^; Additional file 3: Figures S5 and S6), to genes regulated by REST, SOX2, NANOG and OCT4 (*P* < 6.6 × 10^-10^), and to regions with the repressive H3K27me3 mark or bivalent H3K4me3/H3K27me3 marks (*P* < 0.001; Figure 5C).

The methylation levels of each of the relapse DMCs increased in each of the ALL pairs, with the highest levels after the second relapse (Figure 5D). The β-values of the CpG sites in the relapse signature were highly similar in the Nordic Society of Pediatric Hematology and Oncology (NOPHO) and QcALL sample sets (Figure 5E), suggesting that this signature of DMCs is common to relapsed ALL samples, regardless of subtype and treatment protocol. To visualize individual β-value changes in the paired samples, the top 25 ranking DMCs from the relapse signature are plotted in the paired samples (Figure 5F). Regional analysis surrounding CpG sites in each of the top 25 genes showed that nearby CpG sites displayed concordant (increased) methylation changes at relapse (Additional file 3: Figure S7).

### DNA methylation for predicting relapse-free survival in ALL

**Figure 6:**
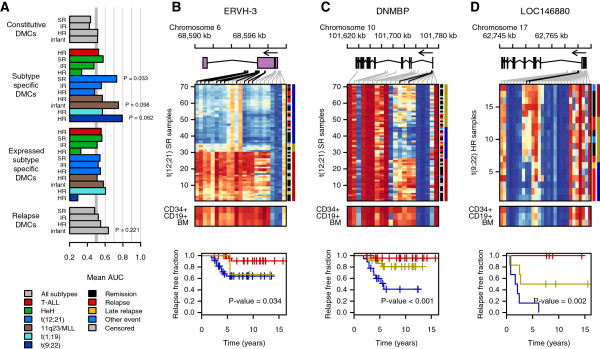
**Prediction of relapse-free survival using differentially methylated CpGs (DMCs). (A)** Performance of the DMC signatures in ALL subtypes stratified according to treatment groups for predicting relapse-free survival using AUC scoring. The treatment groups are indicated by standard risk (SR), intermediate risk (IR), high risk (HR), and infant. The predictive performance of the DMC signatures with AUC >0.60 was assessed by permuting the data 1,000 times. The permuted *P*-values are indicated in the bar chart. Plots of the methylation levels across the **(B)***ERVH-3* and **(C)***DNMBP* genes in patients with the t(12;21)*ETV6/RUNX1* translocation treated with the standard risk protocol and **(D)** the precursor microRNA gene (LOC146880/ENSG00000215769) in patients harboring the t(9;22)*BCR/ABL1* translocation. DMCs associated with relapse-free survival (*P* < 0.05) are highlighted above the heatmaps in **(B-D)** with black lines connecting the CpG site in the gene with the heatmap. The patients (rows) are clustered based on the CpG sites associated with relapse-free survival. The three distinct methylation profiles in the heatmap are indicated by the color bar to the right. The outcome for individual patients is marked by the inner color bar on the right side of the heatmap with patients in remission in black, relapsed patients in red, late relapsed patients in yellow, patients with events other than disease relapse in blue, and patients censored before 5 years of follow-up time in gray. The average methylation levels in the non-leukemic controls are shown below the heatmap. At the bottom of each panel, Kaplan-Meier curves are color-coded by methylation groups, with blue indicating hypomethylation, yellow indicating intermediate methylation, and red indicating hypermethylation. The Kaplan-Meier curves demonstrate the difference in relapse-free survival of patients with different methylation profiles with the Gray’s test *P*-value for the difference shown in each panel.

Finally, we utilized CpG sites that constitute the four signatures of differential methylation defined in this study to search for DMCs that are predictive of relapse-free survival of ALL patients. For this purpose, relapse-free survival in each ALL subtype further stratified into standard risk (SR), intermediate risk (IR), high risk (HR), and infant (I) treatment groups was analyzed against the β-values of the DMCs comprising the constitutive, subtype-specific, subtype-specific correlated with gene expression, and relapse signatures using nearest shrunken centroids classification (Additional file 2: Table S17; Additional file 3: Figure S8) [23]. Four of the methylation signatures allowed for prediction of relapse-free survival with an area under the receiver operating characteristic (ROC) curve (AUC) >0.60 (Figure 6A). After permutation testing, subtype-specific DMCs in the group of ALL patients with the t(12;21) translocation that had been treated according to the standard risk (SR) protocol (n = 71) were found to be associated with relapse (*P* = 0.033). In addition, the subtype-specific sites in patients with the t(9;22) translocation treated on the high risk (HR) protocol (n = 18) and 11q23/*MLL* rearrangements treated on the infant protocol (n = 14) had indicative *P*-values of 0.062 and 0.098, respectively, despite the small number of samples in these groups (Additional file 2: Table S17). The relapse signature in all patients treated according to the infant protocol was not statistically significant (*P* = 0.22).

The effect of each DMC in the relapse-associated signatures was subsequently assessed using permutation testing (Additional file 4). To reduce spurious associations, we required a minimum of two significant CpG sites within the same gene or within 50 kb of each other. Genomic regions were analyzed individually for predictive classification of relapse-free survival. This resulted in the identification of six genomic regions in t(12;21), eight in 11q23/*MLL*, and one in t(9;22), whose methylation values were associated with relapse (Table 3). Strikingly, 11 of the top ranking DMCs for relapse-free survival in the t(12;21) subtype were annotated to a 2.2 kb region on chr6q12, which encodes an endogenous retroviral gene, *ERVH-3*[24] (Figure 6B). In addition, two CpG sites in the *DMNBP* gene distinguished a group of t(12;21) patients with promoter hypomethylation and high risk of relapse (Figure 6C). Two CpG sites in the first intron of the non-coding RNA gene *LOC146880* (ENSG00000215769/hsa-mir-6080) in patients harboring t(9;22) translocations also distinguished a group of patients with hypomethylation and high risk of relapse (Figure 6D). The additional genes associated with increased risk of relapse are plotted in Additional file 3: Figure S9 to S11. These genes include *PAG1* in t(12;21), which is known to harbor recurrent somatic mutations in pediatric ALL patients with the hypodiploid karyotype [25], and *WT1* in *MLL*/11q23, which is commonly mutated in acute myeloid leukemia [26]. Mutations in both these genes are associated with increased risk of relapse in pediatric leukemias. Five zinc finger genes (*ZSCAN18*, *ZNF256*, *ZNF329*, *ZNF544*, and *ZNF681*) on chromosome 19q13 were each independently associated with relapse in 11q23/*MLL* patients, with hypomethylation indicating increased relapse (Additional file 3: Figure S10). These findings indicate that DNA methylation levels of individual genes could be potentially useful as clinical biomarkers in addition to the currently used treatment stratification.

**Table 3:** Gene regions with correlation between methylation level and relapse-free survival in ALL

**ALL subtype**^**a**^	**Gene symbol**	**Gene name**	**Chromosome**	**Number of DMCs**^**b**^
t(12;21)*ETV6/RUNX1*	*ERVH-3*	Endogenous retrovirus group H, member 3	6q12	11
	*C1orf222*	Chromosome 1 open reading frame 222	1p36.33	2
	*KCNA3*	Potassium voltage-gated channel, shaker-related subfamily, member 3	1p13.3	2
	*PAG1*	Phosphoprotein associated with glycosphingolipid microdomains 1	8q21.13	2
	*DNMBP*	Dynamin binding protein	10q24.31	2
	*C11orf52*	Chromosome 11 open reading frame 52	11q23.1	2
*MLL*/11q23	*ZSCAN18*	Zinc finger and SCAN domain containing 18	19q13.43	4
	*ZNF544*	Zinc finger protein 544	19q13.43	4
	*TAPBP/DAXX*	TAP binding protein (tapasin)/death-domain associated protein	6p21.3	3
	*WT1*	Wilms tumor 1	11p13	3
	*ZNF681*	Zinc finger protein 681	19p12	3
	*ADARB2*	Adenosine deaminase, RNA-specific, B2 (non-functional)	10p15.3	2
	*ZNF329*	Zinc finger protein 329	19q13.31	2
	*ZNF526*	Zinc finger protein 526	19q13.31	2
t(9;22)*BCR/ABL1*	*LOC146880*	Pri-miRNA; hsa-mir-6080 (ENSG00000215769)	17q24.1	2

^a^BCP ALL cytogenetic subtypes with subtype-specific differentially methylated CpGs (DMCs) predictive of relapse (*P* < 0.1).

^b^Number of sites in the region associated with relapse-free survival (*P* <0.05).

## Discussion

The 450k BeadChips for DNA methylation analysis are particularly suitable for analysis of large sample sets for which next generation bisulfite sequencing is not yet feasible. In the present study, we examined the methylation status of 435,941 CpG sites to determine the methylation patterns in a large set of samples from patients with childhood ALL at diagnosis (n = 764), relapse (n = 27), and in non-leukemic reference samples (n = 137). The quantitative methylation data from the 450k BeadChips in our large set of ALL samples at diagnosis revealed that the average absolute β-value difference between ALL cells and reference cells for the subtype-specific DMCs is approximately 0.50. Similarly, the β-value difference from pair-wise analysis of ALL cells at diagnosis and at relapse is close to 0.5. Based on these observations we speculate that differential methylation occurs in an allele-specific manner in ALL, analogously to what has been recently suggested by integrative analysis of single nucleotide polymorphisms and methylation using next-generation sequencing in prostate cancer [27]. Our speculation on allele-specific DNA methylation is also substantiated by the quantitative correlation between DNA methylation and allele-specific gene expression that we observed in an earlier study of close to 200 of the diagnostic ALL samples analyzed here [28].

We analyzed multiple cytogenetic subtypes of ALL and found a core methylation signature shared by all the subtypes. This set of 'constitutive' DMCs, which comprised approximately 25% of all DMCs in each ALL subtype, were predominantly hypermethylated and associated with promoters repressed by the polycomb group proteins (PcG) in the context of bivalent chromatin. In stem cells, the repressive PcG complex cooperates with OCT4, SOX2 and NANOG to silence lineage-specific genes and to preserve the pluripotent state of the cells. Hypermethylation preferentially targets CpG islands of PcG-regulated genes in solid cancers [29-31] and in leukemias [13,32,33], which suggests a common signature of hypermethylation across cancer types by which cells lose their plasticity, giving them the ability to differentiate while retaining unlimited self-renewal capacity [31]. Although the expression of the majority of the PcG-regulated genes did not appear to be down-regulated in our data set, other studies [29,31,34] have shown that these genes are usually expressed at very low levels in normal cells, and become fully silenced upon aberrant DNA methylation in cancer cells. In our digital gene expression (DGE) data, the low expression levels of these genes (<0.5 transcripts per million) inhibited accurate quantification of differential expression.

To our knowledge, our study is the first to observe a signature with higher DNA methylation levels of PcG target genes at relapse of ALL than at diagnosis. ALL cells at relapse are generally more resistant to chemotherapeutic treatment, which is consistent with the association between drug resistance and hypermethylation that is beginning to emerge in hematological neoplasms [13,35-37]. Hypermethylation may be reversible by pretreatment with a histone deacetylase inhibitor (vorinostat) and DNA methyltransferase inhibitor (decitabine) before standard chemotherapy [14]. In total, 74 of the genes in the constitutive and/or relapse DMC signatures that we identified in the current study have been experimentally shown to be targets for demethylation by decitabine (*P* < 3.96 × 10^-9^). As recent evidence suggests that cancer cells become dependent on DNA methylation acquired at specific positions [38], targeting the DNA methylation machinery may provide novel treatment options for cancers with hypermethylation phenotypes, especially for those patients who have relapsed [39].

In our study we established that additional hypermethylation in enhancers (marked by H3K4me3/H3K27ac) and in gene bodies are strongly associated with gene expression. Enhancers are distal elements that regulate gene expression and are influenced by aberrant DNA methylation in several cancer types [2,40-42]. We show here that DNA methylation of enhancers is associated with differential gene expression in ALL. We also found that hypomethylation is prevalent outside CpG islands in gene bodies, and can be associated with either increased or decreased gene expression. This observation suggests a complex relationship between methylation in gene bodies in the regulation of gene expression, which may be acting via alternative promoter usage, splicing, and activity of other regulatory elements [40]. Because the regions with histone marks to which DMCs in ALL cells were enriched originated from normal fractionated blood cells [17], our results warrant an investigation of histone marks in primary ALL cells, which like DNA methylation are potentially altered in ALL.

The DNA methylation status of individual candidate genes has been demonstrated to predict clinical outcome and allow refined subgrouping of ALL in a clinical setting [10,43,44]. We utilized the signatures of differentially methylated CpG sites identified in our study to screen for new markers of relapse in ALL, and found that subtype-specific DMCs may be useful as prognostic markers. We detected differential methylation of multiple CpG sites clustered in the *ERHV-3*, *DMNBP*, *KCNA3*, *PAG1*, and *C11orf52* gene regions that were associated with increased risk of relapse in patients with the t(12;21) translocation treated according to standard risk (SR) therapy. In other patient subgroups we did not observe any significant association between DMCs and clinical outcome (*P* < 0.05). Patients with HeH and t(12;21) represent the two largest subgroups in pediatric BCP ALL (Table 1), and a majority of them are stratified to standard risk (SR) therapy. One possible explanation for the lack of DMCs with predictive power in patients with HeH is that this subtype group is less homogeneous than the t(12;21) group, and that various combinations of extra chromosomes in HeH cause differences in treatment response, something we will try to explore further. In all other BCP ALL subgroups, patient numbers were considerably smaller, which hinders analysis by repeated cross-validation. As in other contemporary ALL protocols, the current NOPHO ALL2008 protocol includes more intense treatment with asparaginase for all patients than the earlier treatment protocols that were used for the patients included in this study [45]. When follow-up times are long enough, it will be interesting to see if the same genes continue to have prognostic significance for patients treated on the most recent NOPHO ALL2008 protocol. Several studies have reported cancer-associated hypomethylation, expression, and a link to poor outcome for some of the human endogenous retrovirus families [46]. Although hypomethylation or expression of *ERVH-3* has not previously been associated with outcome in t(12;21) BCP ALL, this gene was originally discovered in the REH ALL cell line bearing the t(12;21) translocation [24]. A recent study in acute myeloid leukemia showed that decitabine treatment of acute myeloid leukemia cells causes hypomethylation and up-regulation of *ERVH-3* expression [47]. Our findings of hypomethylation in the *ERVH-3* gene as a marker of relapse in t(12;21) warrant exploration of the side effects of decitabine treatment on abnormal hypomethylation of endogenous retroviral genes.

## Conclusions

We generated a comprehensive view of the methylation landscape in pediatric ALL compared to non-leukemic reference cells. The analysis identified prevalent hypermethylation of CpG sites at diagnosis and relapse in all subtypes of pediatric ALL. We also detected discrete differences in methylation that drives differential gene expression in a subtype-specific pattern. Moreover, hypomethylation of several genes appeared to be predictive of relapse in a subset of patients with the common t(12;21)*ETV6/RUNX1* translocation. Whether the *de novo* methylation detected here contributes actively to ALL, or is a passenger in the malignant transformation of blood progenitor cells into ALL cells remains to be elucidated. Our study implies that aberrant DNA methylation is a signature of leukemic development and progression, and for the heterogeneity between patients of similar cytogenetic backgrounds that contributes to relapse.

## Materials and methods

### DNA and RNA samples

BM aspirates or peripheral blood samples were collected from pediatric ALL patients enrolled in the NOPHO ALL92 or ALL2000 protocols [5]. Clinical follow-up data were obtained from the NOPHO registry. The median follow-up time for patients in continuous complete remission was 9.1 years (range 4.6 to 18 years). Lymphocytes were isolated from ALL samples at diagnosis (n = 764), remission (n = 86), first relapse (n = 27), and second relapse (n = 5) by Ficoll-isopaque centrifugation (Pharmacia, Uppsala, Sweden; Table 1). All samples included in the study contained >80% leukemic blasts at diagnosis (average 91%) and relapse (average 90%), and <5% at remission. For validation, a sample set of DNA samples that were isolated at diagnosis, remission, and relapse from 10 children with pediatric BCP ALL from the QcALL cohort was used. Clinical information for QcALL and relapse samples is available in Additional file 2: Table S15. CD19+ B cells and CD3+ T cells were isolated from peripheral blood mononuclear cells of healthy Swedish blood donors using positive selection (CD19 Microbeads #120-050-301 and CD3 Microbeads #130-050-101) and MACS cell separation reagents (Miltenyi Biotec, Bergisch Gladbach, Germany). Pooled CD34+ cells isolated from five healthy blood donors were purchased from 3H Biomedical (Uppsala, Sweden) [48]. DNA and RNA were extracted as previously described [19,28]. The study was approved by the Regional Ethical Review Board in Uppsala, Sweden and was conducted according to the guidelines of the Declaration of Helsinki. The patients and/or their guardians provided informed consent.

### DNA methylation assay

DNA was treated with sodium bisulfite (EZ DNA methylation Gold, Zymo Research, Irvine, CA, USA) and DNA methylation levels were measured using the Infinium HumanMethylation 450k BeadChip assay (Illumina, San Diego, CA, USA). The ALL samples and controls were randomly distributed across the arrays, all arrays were measured using the same HiScan instrument, and no evidence for batch effects was observed in the β-values (data not shown). The methylation β-value distribution between Infinium type I and II probes was normalized using peak-based correction (Additional file 3: Figure S12) [49]. The data were filtered by removing the data from probes on the X and Y chromosomes and with genetic variation affecting probe hybridization (Additional file 3: Figure S13). After filtering, methylation data for 435,941 CpG sites remained for further analysis (Additional file 1). A subset of diagnostic ALL samples (n = 364) were previously analyzed on a custom GoldenGate DNA methylation array (Illumina) [10]. DNA methylation values of 207 CpG sites interrogated by both arrays evaluate reproducibility of the β-value measurements (Additional file 3: Figure S14). Additional details about the methylation assay, probe filtering, and technical validation can be found in Additional file 4. The DNA methylation data are available at the Gene Expression Omnibus (GEO) with accession number GSE49031.

### Annotation of CpG sites

CpG sites were annotated to RefSeq genes and CpG islands according to the Human Methylation 450k manifest file version 1.1. The distribution of probes that passed our stringent filtering is shown in relation to CpG islands, gene regions, and corresponding β-value distributions are shown in Additional file 3: Figures S15 and S16. When a CpG site had more than one gene-level annotation, that is, was present in both the transcription start site and the first exon, both annotations were used.

The following publicly available chromatin datasets from primary CD19+, CD3+, or CD34+ cells were obtained from the NIH Roadmaps Epigenomics Project: DHS regions, H3K27me3, H3K36me3, H3K4me3, H3K9me3, and H3K4me1 (Additional file 2: Table S18) [17]. Peaks were called using the MACS software using default settings [50]. H3K27ac peaks were downloaded from the UCSC table browser [51] derived from H1-hESC and GM12878 cell lines [18]. CpG sites were annotated for the chromatin marks by overlapping genomic location with a peak in at least two of the replicates analyzed (Additional file 2: Table S1).

### Analysis of differential DNA methylation

DMCs were determined using the non-parametric Wilcoxon rank-sum test. They were determined in T-ALL using remission BM, CD3+, and CD34+ cells as reference and in BCP ALL using remission BM, CD19+, and CD34+ cells. The Wilcoxon signed-rank test was used to identify methylation differences between paired samples at diagnosis and relapse. Minimal cut-off values for the mean absolute differences in DNA methylation (∆β) of 0.2 were applied to highlight CpG sites with large differences between groups. CpG sites with standard deviations >0.10 in the reference control group (n = 33,533 sites) were removed from DMC lists to minimize DMCs occurring due to cell type-specific variability (Additional file 3: Figure S2).

### Correlation between DNA methylation and gene expression

Genome-wide digital mRNA gene expression (DGE) sequencing data from 28 ALL patient samples and five non-leukemic reference samples were generated as previously described (Additional file 2: Table S4) [19]. RNA expression levels for 93 ALL patient samples were measured with Affymetrix U1333 Plus 2.0 arrays (Additional file 2: Table S6). Raw data were processed and normalized using the robust multichip average (RMA) algorithm [19,52]. The expression datasets are publicly available at GEO under series GSE47051. Details on the gene expression assays can be found in Additional file 4. For each DMC signature, the correlation between β-value and log2 transformed gene expression was evaluated using the Pearson’s correlation coefficient. Statistical significance of each DMC was calculated by permuting the data 10,000 times and comparing the correlation coefficient in the unpermuted data to the permuted coefficients. In each dataset, the permuted *P*-values were adjusted for multiple testing using the Benjamini and Hochberg approach for controlling FDR [53].

### Data analysis and visualization

Data analysis was carried out in the R environment [54]. The R code for the analyses performed in this study is available at GitHub [55]. One-sided Fisher’s exact tests were used to assess the significance of the enrichment of DMCs to functionally annotated regions, using the annotation of the 450k array as background. Pathway analysis and enrichment for upstream regulators was performed using software from Ingenuity Pathway Analysis (Ingenuity® Systems, Redwood City, CA, USA) and significance was evaluated with the Fisher’s exact test. All *P*-values were adjusted for multiple testing by FDR unless otherwise stated. Analysis of relapse-free survival for constitutive and relapse DMC signatures was performed on all patients. Relapse-free survival for the subtype-specific signatures was evaluated individually for T-ALL and BCP ALL separated into the cytogenetic subtypes 11q23/*MLL*, HeH, t(1;19), t(12;21), and t(9;22). Each subtype was further stratified according to standard, intermediate, high risk, or infant treatment protocols [5]. The patients with dic(9;20) and iAMP21 were not analyzed for relapse-free survival due to the small number of patients in each treatment group. Nearest shrunken centroids classifiers were designed to discriminate between the classes and evaluated with repeated cross-validation [23]. AUC was used to measure predictive performance and statistical significance was evaluated by permuting the data 1,000 times. Each CpG site was scored by its coefficient after shrinkage and the significance was evaluated by permutation testing, as described above. Further details on the relapse-free classification procedure can be found in Additional file 3: Figure S8 and Additional file 4.

## Abbreviations

ALL: acute lymphoblastic leukemia; AUC: area under the ROC curve; BCP ALL: B-cell precursor acute lymphoblastic leukemia; BM: bone marrow; DHS: DNase1 hypersensitivity; DMC: differentially methylated CpG site; GEO: Gene Expression Omnibus; HeH: high hyperdiploid; NOPHO: Nordic Society of Pediatric Hematology and Oncology; PCA: principal component analysis; QcALL: Quebec childhood ALL; ROC: receiver operating characteristic; T-ALL: T-cell acute lymphoblastic leukemia; UTR: untranslated region.

## Competing interests

The authors declare that they have no competing interests.

## Authors’ contributions

ACS, JN, and GL designed the study. GL coordinated clinical sample procurement. EF provided expertise on patient karyotypes. TF, EF, BMF, MH, AHS, RL, KS, SS, and GL provided samples and clinical information. SB, DS, and TP provided the validation cohort. MLE and LR provided control samples. PW and ECB provided expertise on genomic analyses. MGG supervised multivariate data analyses. JN and CLB performed the bioinformatics and statistical analyses. JN, ACS, CLB, PW, and GL wrote the paper. All authors read and approved the final manuscript.

## Supplementary Material

Additional file 1: Table S1 (Tab delimited .txt) Probe-level annotations for the 485,577 probes on the 450k array, including a column denoting the 435,941 CpG sites analyzed in the current study. Columns representing the differentially methylated CpG (DMC) signatures are included. Click here for file

Additional file 2: Tables S2 to S18 Supplemental Tables S2 to S18. Click here for file

Additional file 3: Figures S1 to S16 Supplemental Figures S1 to S16. Click here for file

Additional file 4 Supplemental materials and methods. Click here for file
