# Supplementary material for: Genome-wide signatures of differential DNA methylation in pediatric acute lymphoblastic leukemia
Source: Genome Biol. 2013 Sep 24;14(9):r105. doi: 10.1186/gb-2013-14-9-r105 (PMC4014804; doi:10.1186/gb-2013-14-9-r105)

**Supplemental Figures**

**Genome-wide signatures of differential DNA methylation in pediatric acute lymphoblastic leukemia**

Jessica Nordlund, Christofer L. Bäcklin, Per Wahlberg, Stephan Busche, Eva C. Berglund, Maija-Leena Eloranta, Trond Flaegstad, Erik Forestier, Britt-Marie Frost, Arja Harila-Saari, Mats Heyman, Ólafur G. Jónsson, Rolf Larsson, Josefine Palle, Lars Rönnblom, Kjeld Schmiegelow, Daniel Sinnett, Stefan Söderhäll, Tomi Pastinen, Mats G. Gustafsson, Gudmar Lönnerholm & Ann-Christine Syvänen

**Figure S1.** **Differentially methylated CpG sites (DMCs) in acute lymphoblastic leukemia (ALL) cells.** (A-F) The difference in methylation β**-**values of the 9,406 constitutive differentially methylated CpG sites (DMCs) between the non-leukemic and ALL cells. In each panel, the mean methylation value for each DMC is plotted in non-leukemic controls (left) and ALL cells (right). Each CpG site is connected by a solid line, red for DMCs hypermethylated in ALL and blue for DMCs hypomethylated in ALL. The dashed lines represent the average methylation level across all hyper- and hypomethylated DMCs, respectively. (A) The panel of non-leukemic reference control samples for determining DMCs in BCP-ALL subtypes consisted of CD19+ B-cells, CD34+ hematopoietic stem cells, and mononuclear cells isolated from bone marrow (BM) of pediatric ALL patients in remission. (B) The panel of non-leukemic control samples used to determine DMCs in the T-ALL subtype consisted of CD3+ T-cells, CD34+ cells, and healthy BM as described above. (C) The mean methylation differences between the non-leukemic BM and ALL cells. (D) The mean methylation differences between the DMCs in CD19+ B-cells and ALL cells. (E) The mean methylation differences for the DMCs in CD3+ T-cells and ALL cells. (F) The methylation differences between the β-values of the DMCs in the CD34+ sample and mean β-values in ALL cells.


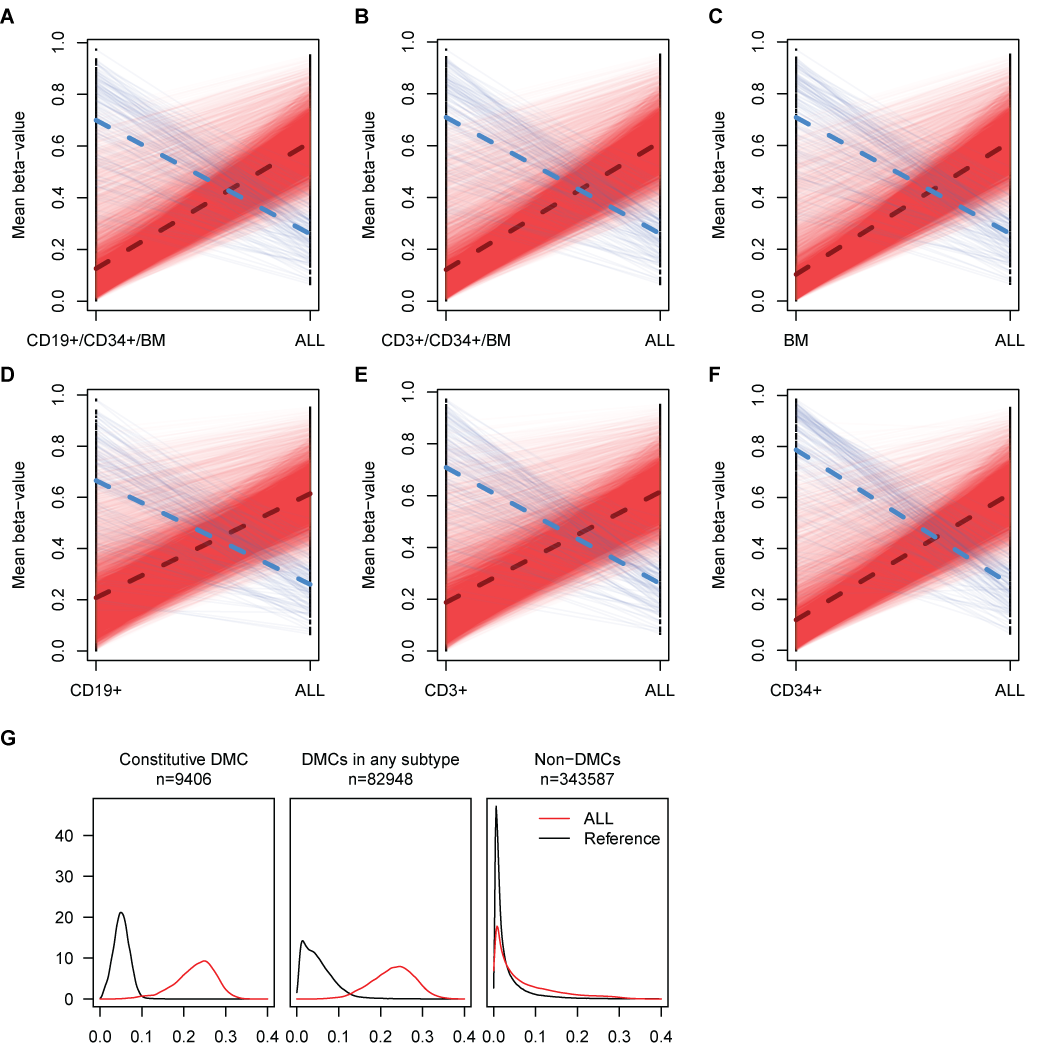


**Figure S2**. Variance in DNA methylation β-values in acute lymphoblastic leukemia (ALL) samples and non-leukemic reference samples according to their functional genomic location. The intra-group variance of CpG sites in acute lymphoblastic leukemia samples (left) and non-leukemic reference samples (right) are plotted. The variance of probes (A) by relationship to CpG islands and (B) by relationship to gene region annotations are plotted with frequency of observations as a function of standard deviation (top panels) and box plots with the standard deviations in the annotation classes on the vertical axis.


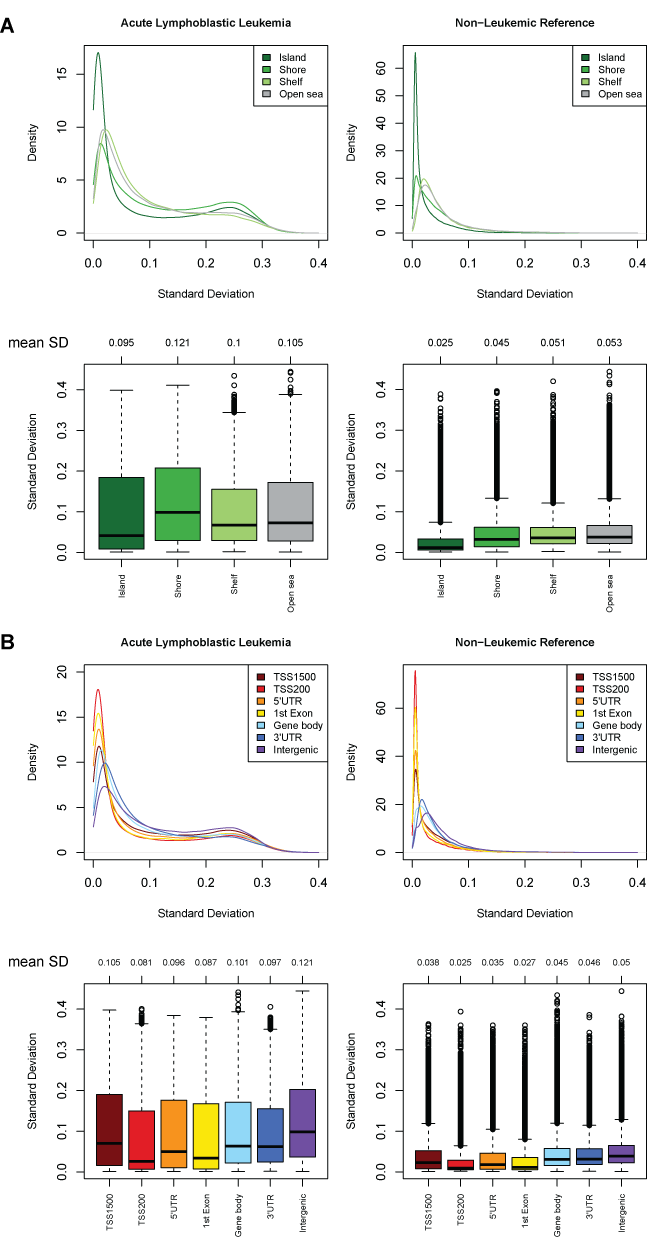


**Figure S3.** Enrichment tables for functional genomic locations of the DMCs that are correlated with gene expression in ALL subtypes. In each panel, the number of subtype-specific hypermethylated (red) and hypomethylated (blue) CpG sites that correlate with decreased (-) or increased (+) gene expression (FDR adjusted permuted p-value <0.05 and fold change >2) are plotted by functional annotation. Fold enrichment for each annotation was calculated for DMCs correlated with gene expression in comparison to all DMCs in that subtype. Bolded numbers indicate annotations with significant enrichment (Bonferroni corrected one-sided Fisher’s exact p<0.001).


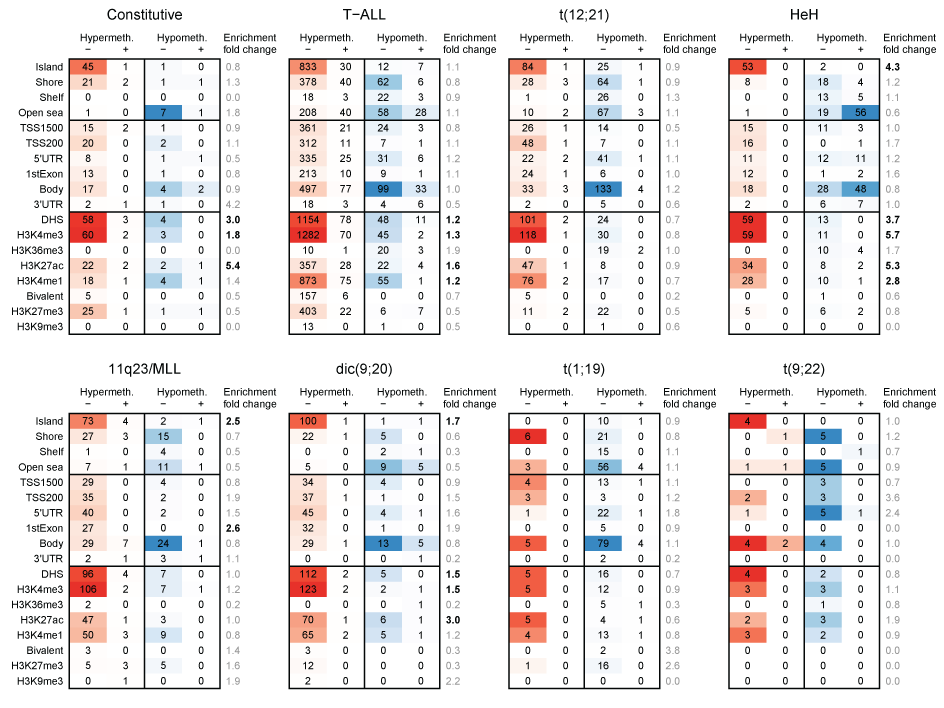


**Figure S4.** Principal component analysis (PCA) of samples from acute lymphoblastic leukemia (ALL) patients at diagnosis and relapsed based on the genome-wide DNA methylation data performed independently for each class of annotated sites. In each panel the first two components from the PCA highlight the methylation patterns of the paired samples are indicated by connected solid lines. Diagnostic samples are color coded in yellow, first relapse in red, and second relapse in purple. The last relapse sample in each pair is indicated with an arrow. (A) PCA of CpG sites in CpG islands, (B) in “shores”, (C) in “shelves”, and (D) in “open sea”.


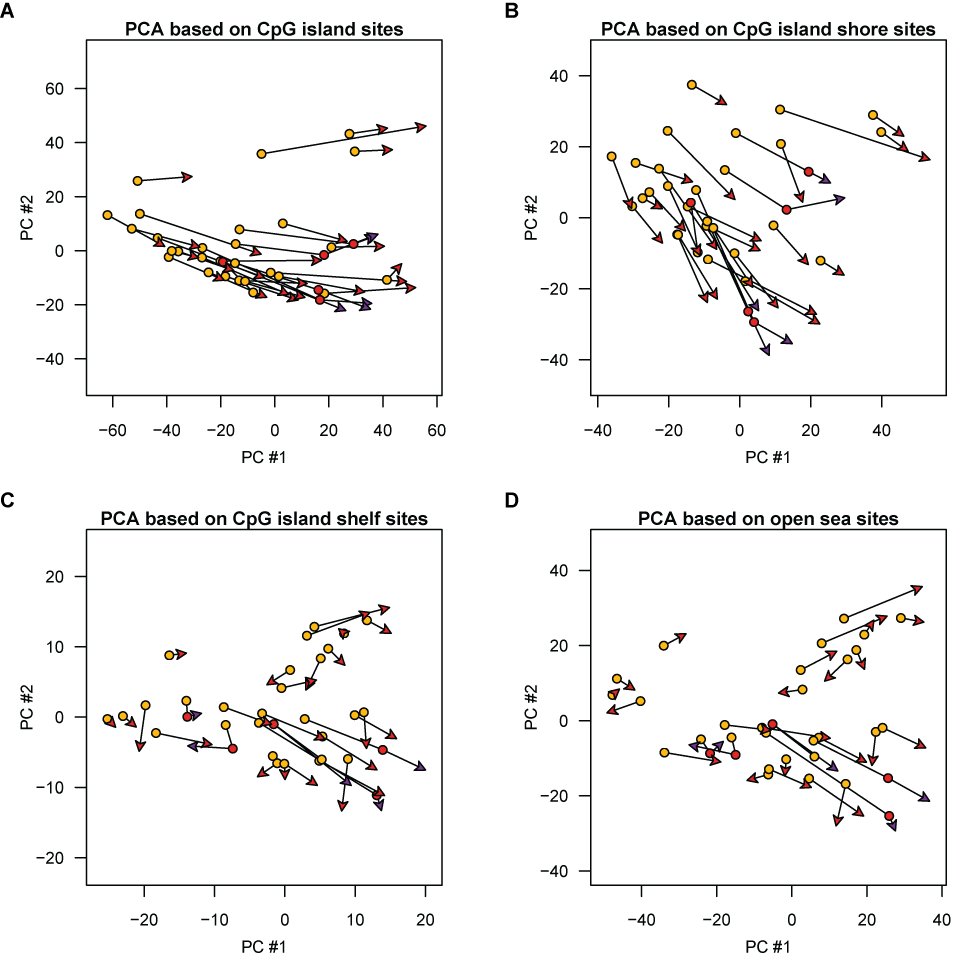


**Figure S5.** Schematic drawing of the genes involved in the transcriptional regulatory network in embryonic stem cells canonical pathway in the Ingenuity Knowledge Base. Genes with significant differential methylation at diagnosis, relapse, and genes that were significant in both analysis are highlighted.

**
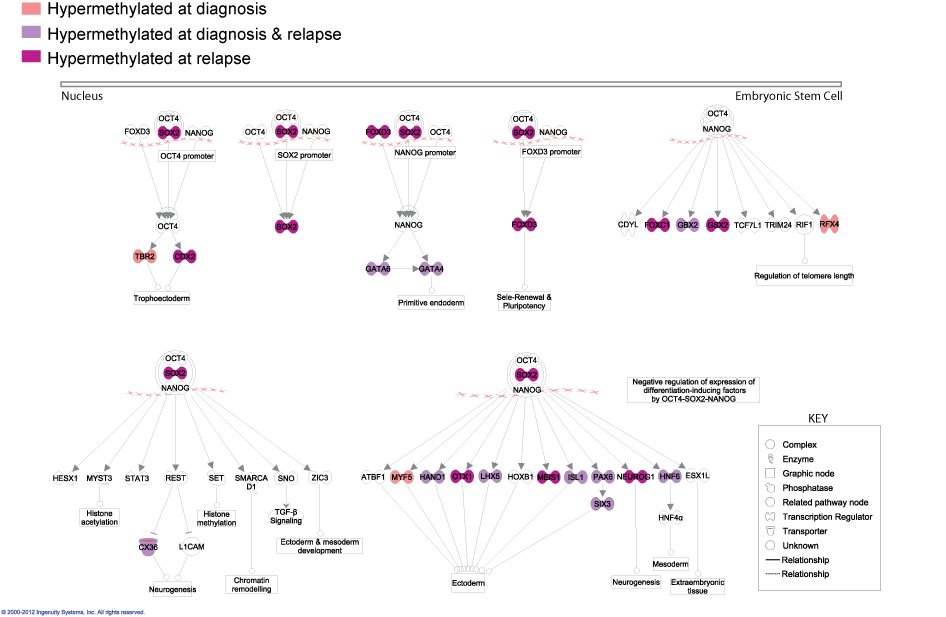
**

**Figure S6.** Schematic drawing of the genes Wnt/β-catenin signaling canonical pathway in the Ingenuity Knowledge Base. Genes with significant differential methylation at diagnosis, relapse, and genes that were significant in both analyses are highlighted.

**^
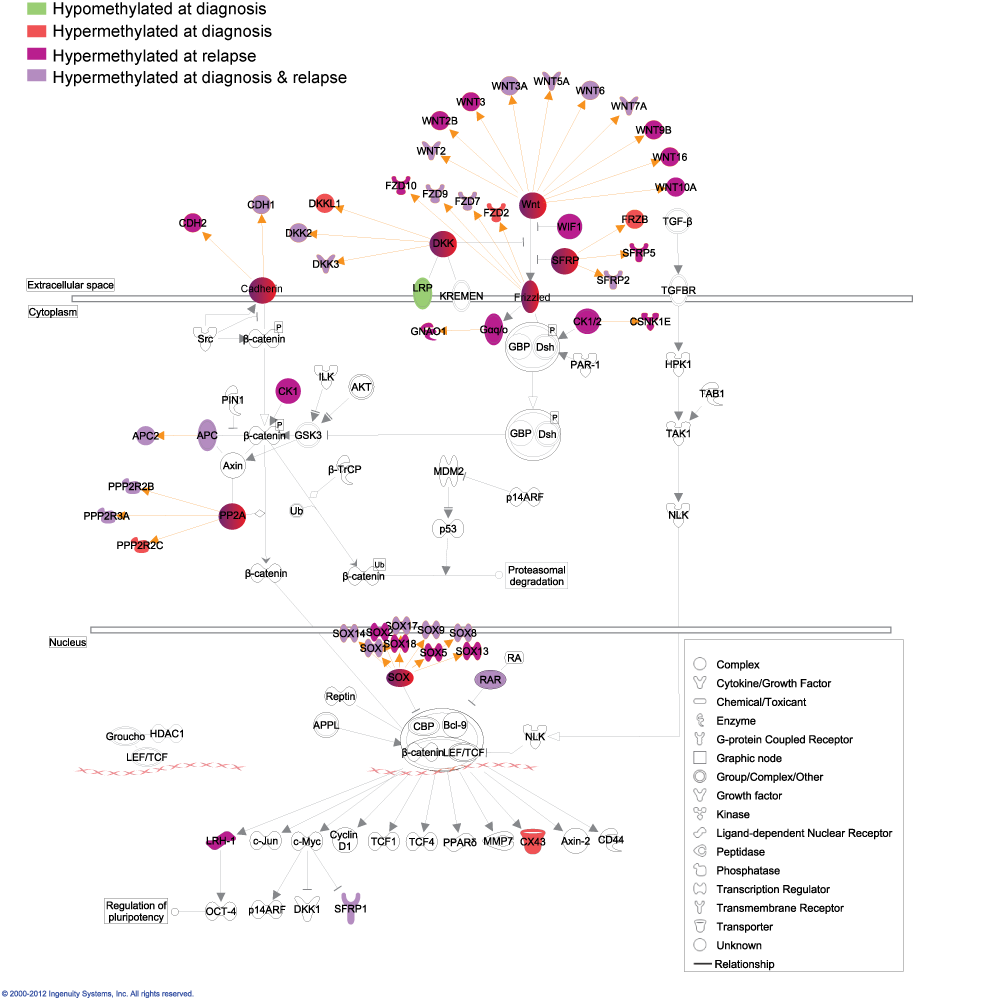
­­­­^**

**Figure S7.** Gene plot heatmaps of the DNA methylation levels of the genes with top ranking DMCs at relapse of acute lymphoblastic leukemia (ALL). For each gene, the canonical transcript is plotted with an arrow indicating the direction of transcription. Where the DMC was between two genes, both are plotted. Vertical boxes indicate exon position. Each CpG site measured by the 450k array and annotated to the gene of interest is indicated with a line connecting the position in the gene to the heatmap below. The top ranking DMC is indicated by a black line. The mean methylation level across the non-leukemic reference samples (CD34+, bone marrow, CD19+ and CD3+) and diagnostic, 1^st^ relapse, and 2^nd^ relapse ALL samples are shown in rows. Blue color indicates sites with low mean methylation and red indicates sites with high mean methylation. CpG islands, shores, and shelves are shown below each heatmap by dark green, green, and light green boxes, respectively.

**
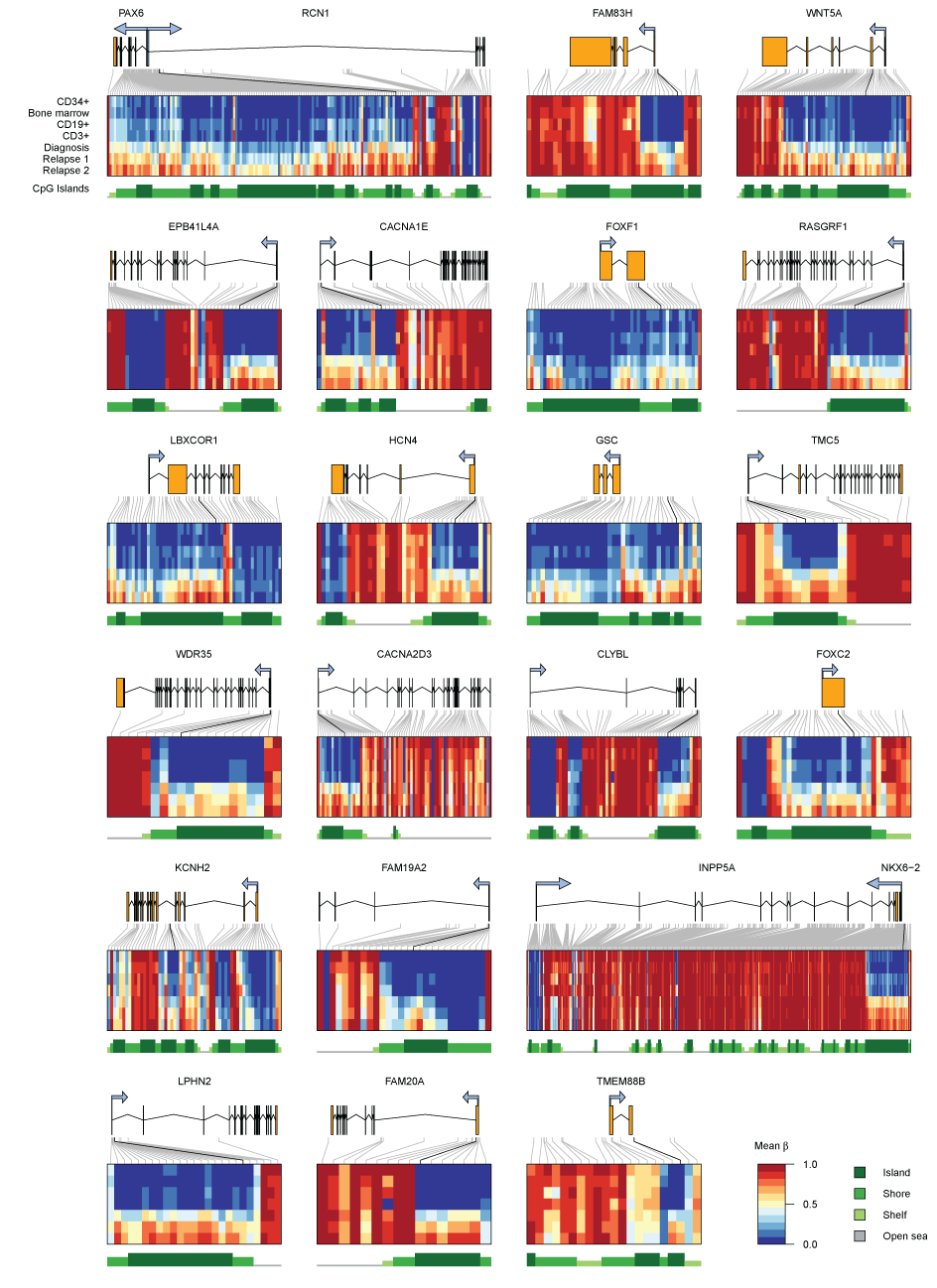
**

**Figure S8.** Flow chart of relapse-free survival modeling procedure. The innermost layer (green) produces the models and predictions, the middle layer (yellow) estimates the performance, and the outmost layer (purple) assesses significance. Further details on the modeling procedure can be found in the supplemental methods (**Additional file 4**).

**
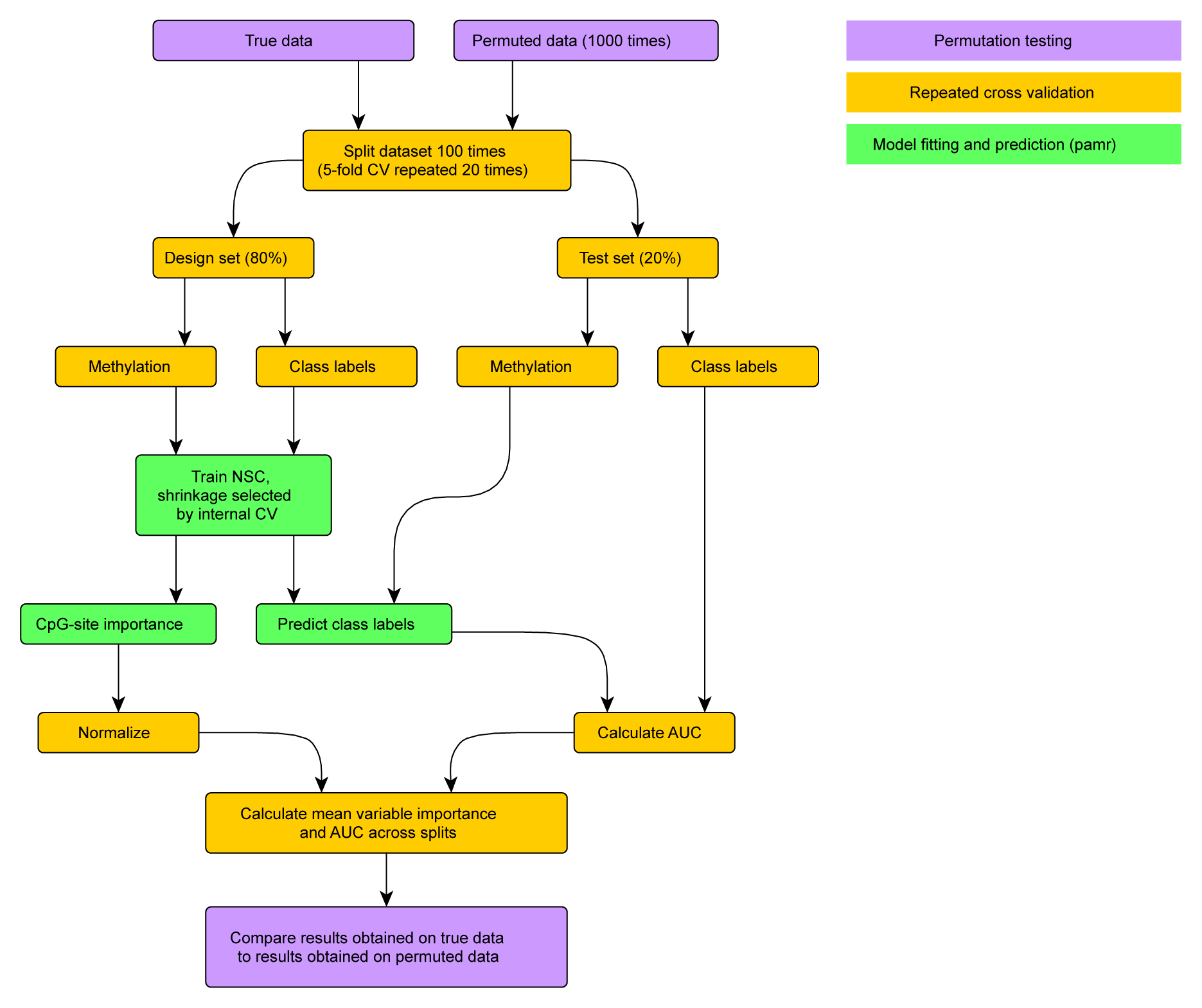
**

**Figure S9.** Genes with at least two significant DMCs (permuted p-value <0.05) associated with relapse-free survival in patients with the t(12;21)ETV6/RUNX1 translocation. For each gene, the canonical transcript is plotted with an arrow indicating the direction of transcription. Vertical boxes indicate exon positions. Each CpG site measured by the 450k array and annotated to the gene of interest is indicated with a line connecting the position in the gene to the heatmap below. The significant DMCs are indicated by black lines. The samples were clustered based on the significant DMCs and split into three groups indicated by the right color strip (blue, brown, red). The outcomes of the patients in the heatmap are shown in the left color strip (black, grey, blue, red, yellow) and the outcomes of the clustering groups are shown in the bottom Kaplan-Meier plots. Uncorrected p-values from the Gray’s test are given.

**
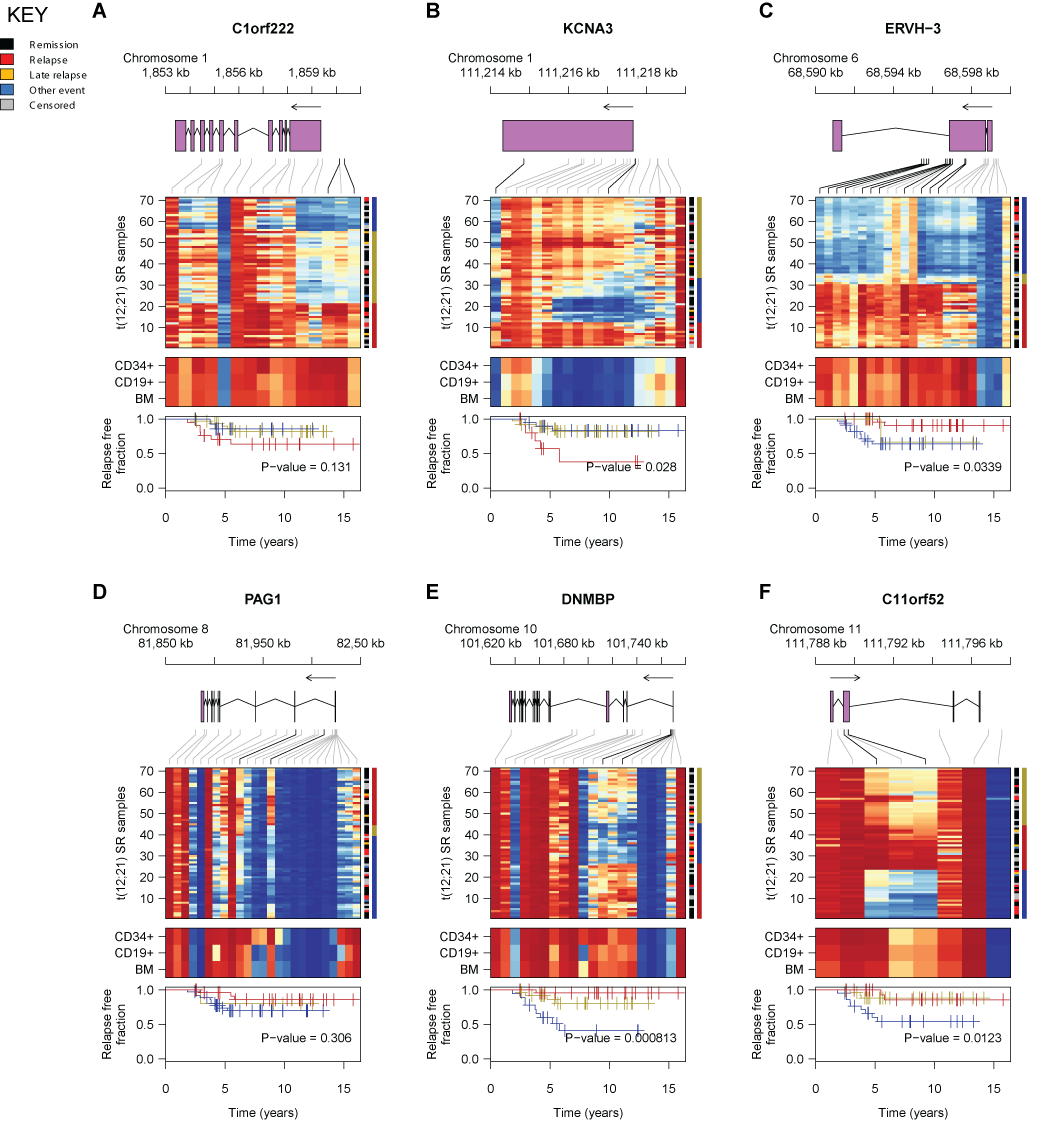
**

**Figure S10.** Genes with at least two significant DMCs (permuted p-value <0.05) associated with relapse-free survival in patients with MLL-rearrangements. See the figure legend for Figure S9 for a description. Due to the small number of samples in this group, they were clustered based on the significant DMCs and split into two groups rather than three, as indicated by the right color strip (blue, red).

**
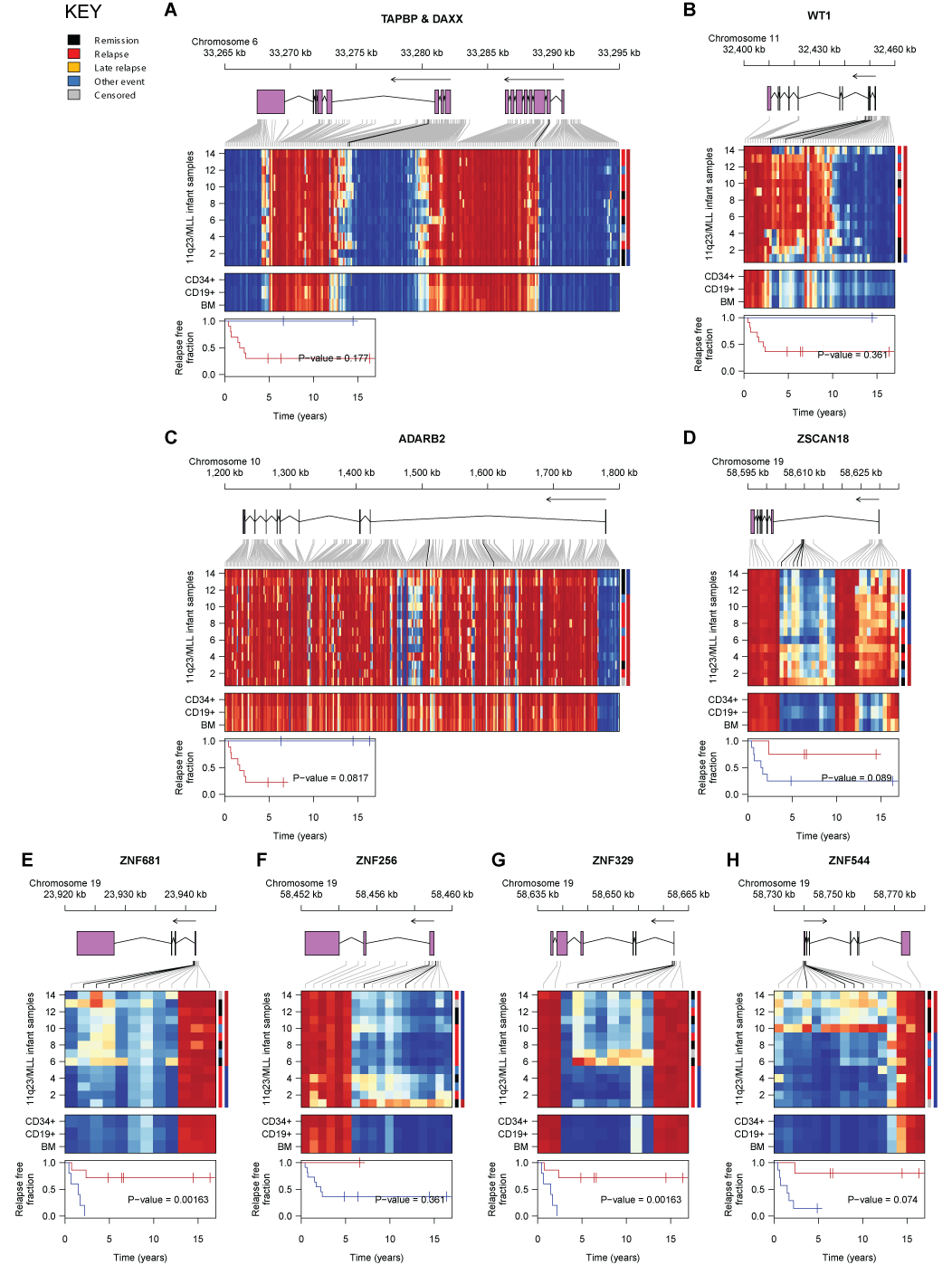
**

**Figure S11.** The non-coding RNA gene, LOC146880 with two significant DMCs (permuted p-value <0.05) associated with relapse-free survival in patients with t(9;22)*ETV6/RUNX1*. See the figure legend for Figure S9 for a description. Due to the small number of samples in this group, they were clustered based on the significant DMCs and split into two groups rather than three, as indicated by the right color strip (blue, red).

**
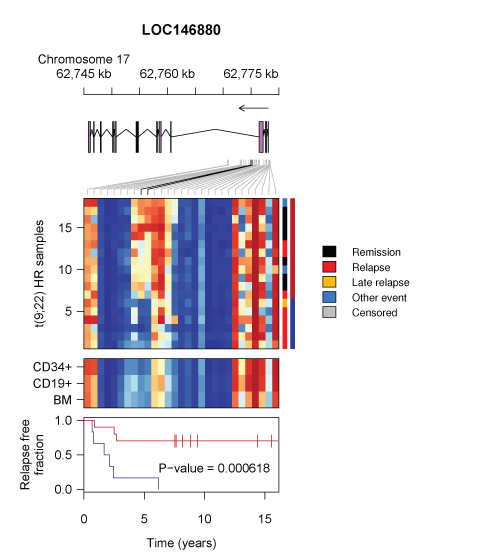
**

**Figure S12**. Methylation β-value distribution of the type I and II probes from all samples included in the study run on the Infinium assay. Type I probes are indicated as a solid black line. Type II probes before peak-based normalization (red dotted line) and type II probes after peak-based normalization (blue dotted line). The figure shows that after peak-based normalization for differences in fluorescence intensities due to the dual color detection, the type II probe distribution is similar to that of the type I distribution. Density is shown on the vertical axis and β-value is shown on the horizontal axis.


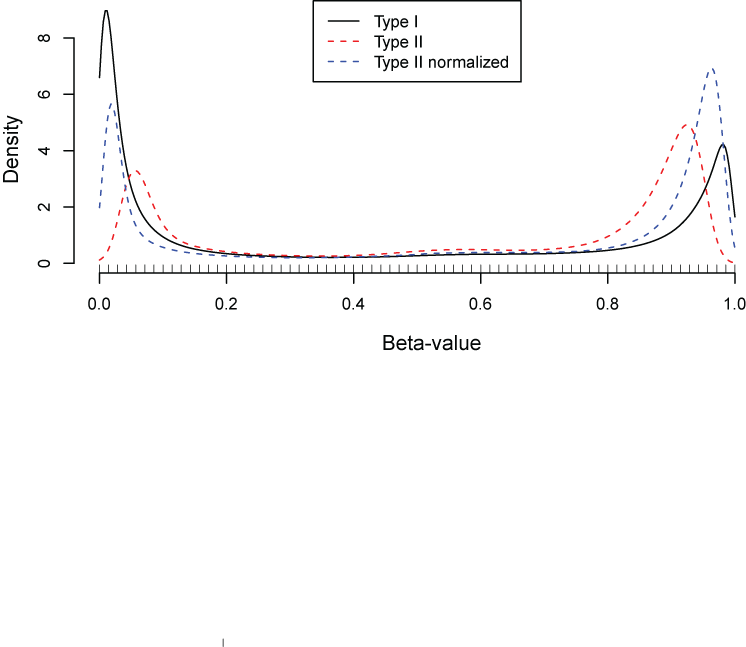


**Figure S13.** Probes that align to multiple places in the genome or overlap genomic regions with polymorphisms display significantly higher variability in DNA methylation β-values. The standard deviation (SD) for each CpG site was calculated across bone marrow aspirates from 86 pediatric ALL patients at remission. (A) The probe sequences were aligned to the human genome build 37 with BWA. Probes mapping to multiple sites were indicated by a BWA mapping score <37. In 200 iterations, the standard deviation (SD) across 1,000 probes that mapped to multiple sites and 1,000 probes mapping to a single site were analyzed for difference in variance with a one sided Wilcoxon rank-sum test (red dots). The test was also performed by comparing 1,000 randomly selected probes mapping to a single site to an additional 1,000 randomly selected probes mapping to a single site (black dots). The result of each test is plotted along the x-axis. The p-values are plotted on the y axis. Points falling above the red horizontal line indicate significant differences in variability between multiple mapping probes and single mapping probes. (B) The SD of probes with annotated SNPs (based on dbSNP 135) at different base positions from the 3’ end of each probe was measured across the same 86 remission samples as in panel A. 1,000 probes from each probe class were randomly chosen and the SD of the 1,000 SNP containing probes was tested against 1,000 probes without SNPs in their binding sites with the wilcoxon rank-sum test. The SD of probes with SNPs in the CpG site in the case of type II probes or the interrogation site in the case of type I probes (red), the 1^st^ base pair (orange), and 2^nd^ base pair (yellow) from the 3’ end of the probe displayed higher variability in β-values than probes without annotated SNPs.


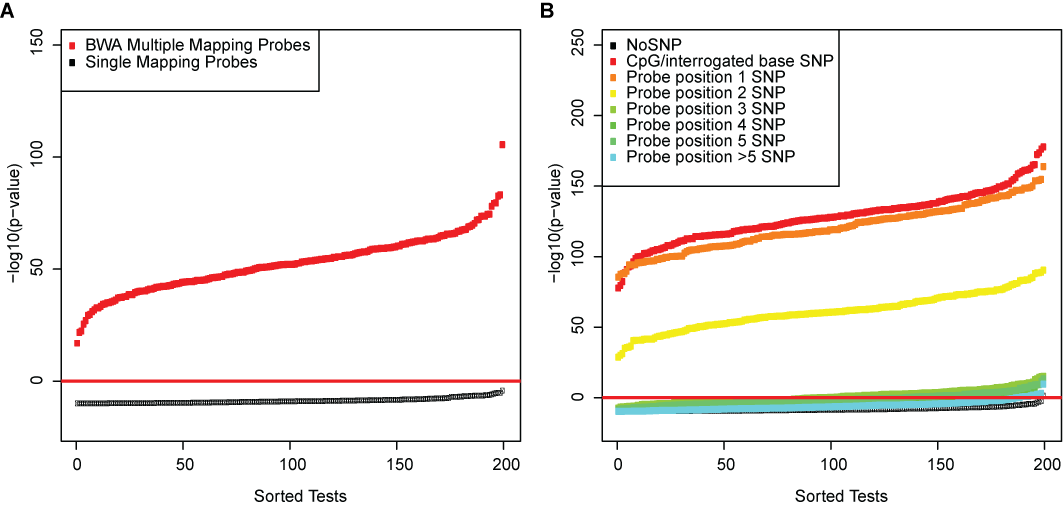


**Figure S14.** Validation of the 450k Methylation Array. (A) Methylation β-values across 207 CpG sites in 364 diagnostic acute lymphoblastic leukemia (ALL) samples measured with the 450k Methylation Array and a custom GoldenGate Methylation Assay (Illumina Inc) (R = 0.92). (B) The β-values of four randomly chosen CpG sites in 364 ALL patients measured by both arrays. (C) Technical replicates (same DNA sample) analyzed twice on the 450k array. (D) Independent bone marrow/peripheral blood samples taken from the same patient at different time points serve as biological replicates for the 450k array. (C-F) Based on analysis of variability in two technical and three biological replicates, we estimated that a delta-β value of >0.05 can be detected with ~90% confidence, a ∆β-value of >0.10 can be detected with 98% confidence, and a ∆β-value of >0.20 can be detected with over 99% confidence.


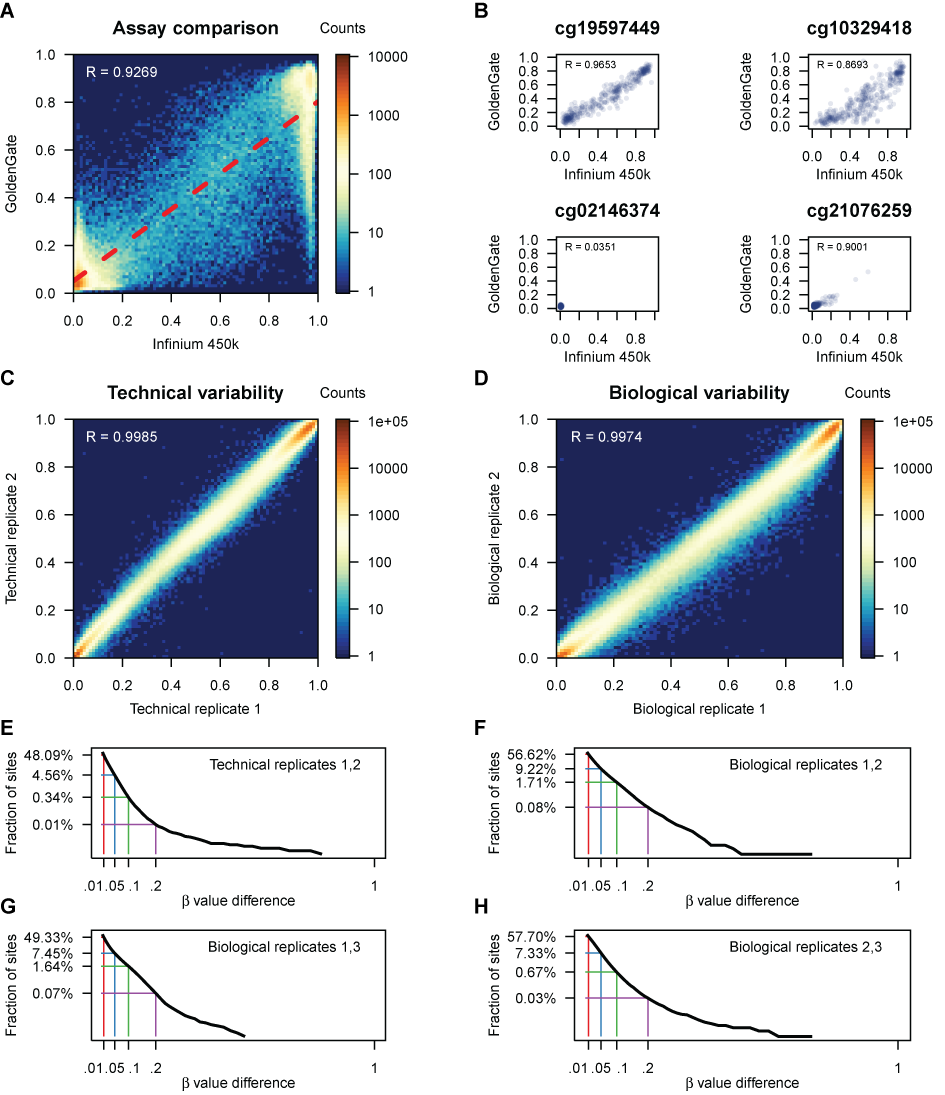


**Figure S15**. The distribution of the 435,941 CpG sites that pass quality filtering. (A) Distribution of CpG sites according to their CpG island relation. Shores are defines as the 0-2kb sequence flanking annotated CpG islands. Shelves are defined as regions within 2-4kb flanking annotated CpG islands. (B) Distribution of CpG sites according to gene regions with promoter regions as -1,500 base pairs (bp) from the transcription start site (TSS1500), -200 bp from the TSS (TSS200), 5’ untranslated region (5’UTR), 1^st^ exon (1stExon), exonic (minus 1^st^ exon) and intronic comprising the gene body, the 3’ untranslated region (3’UTR), and CpG sites not annotated to genes (intergenic). CpG sites can be annotated to more than one gene region depending on transcript isoforms and overlapping genes, thus a CpG site can be assigned more than one annotation and each unique annotation is taken into account per CpG site.


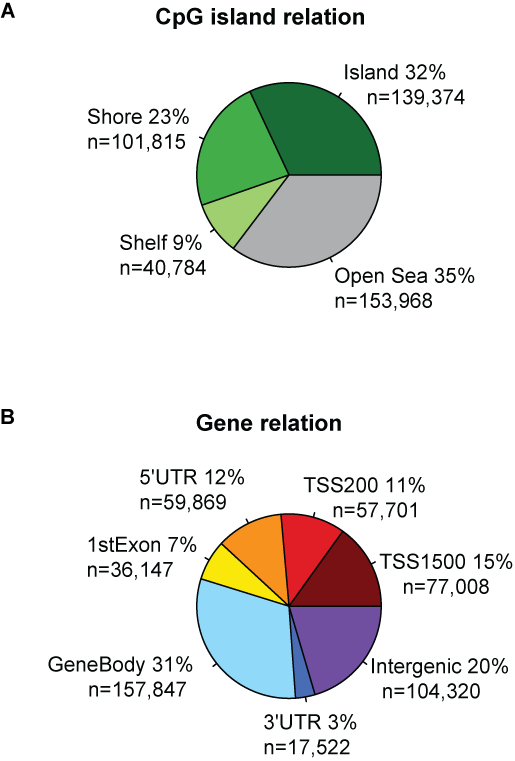


**Figure S16**. Violin plots showing the bimodal distribution of methylation β-values for 435,941 CpG sites. (A-B) Positions of CpG sites plotted in relation to CpG islands in acute lymphoblastic leukemia (ALL) cells and non-leukemic reference samples. (C-D) Positions of CpG sites plotted by gene region annotation in ALL cells and non-leukemic reference samples. Mean β-values are plotted on the x-axis, with the median indicated by white boxes in the violins.


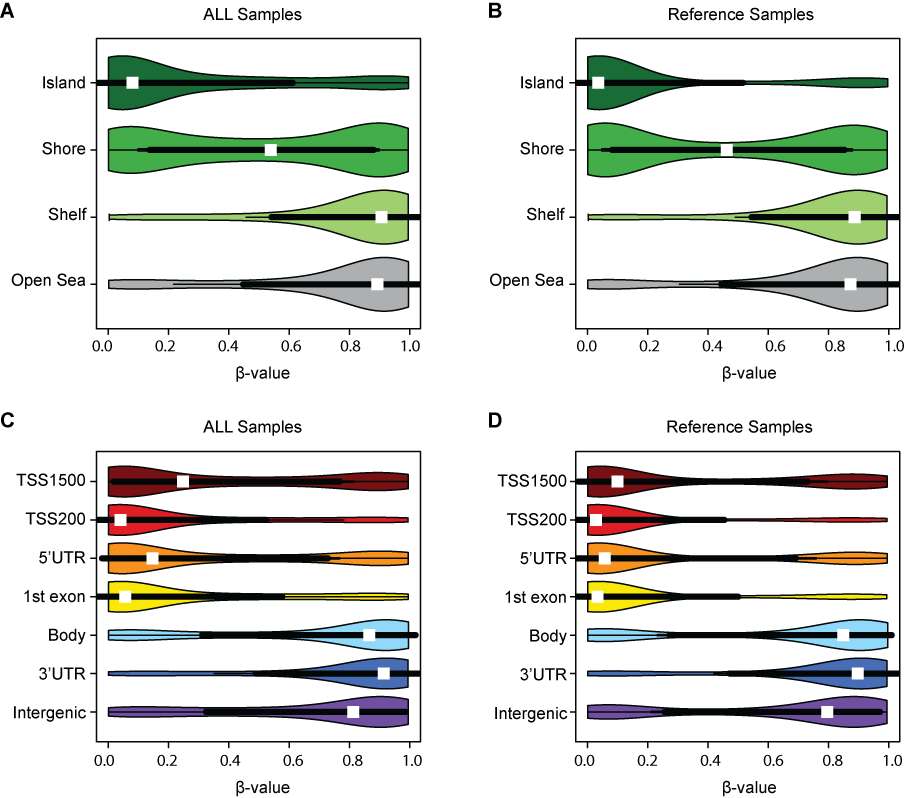

Supplement: Additional file 3: Figures S1 to S16 — Supplemental Figures S1 to S16. [file gb-2013-14-9-r105-S3.docx]
