# Supplementary material for: Genome-wide signatures of differential DNA methylation in pediatric acute lymphoblastic leukemia
Source: Genome Biol. 2013 Sep 24;14(9):r105. doi: 10.1186/gb-2013-14-9-r105 (PMC4014804; doi:10.1186/gb-2013-14-9-r105)
Supplement: Additional file 4 — Supplemental materials and methods. [file gb-2013-14-9-r105-S4.docx]

**Supplemental Materials and Methods**

**Genome-wide differential DNA methylation signatures in pediatric acute lymphoblastic leukemia**

Jessica Nordlund, Christofer L. Bäcklin, Per Wahlberg, Stephan Busche, Eva C. Berglund, Maija-Leena Eloranta, Trond Flaegstad, Erik Forestier, Britt-Marie Frost, Arja Harila-Saari, Mats Heyman, Ólafur G. Jónsson, Rolf Larsson, Josefine Palle, Lars Rönnblom, Kjeld Schmiegelow, Daniel Sinnett, Stefan Söderhäll, Tomi Pastinen, Mats G. Gustafsson, Gudmar Lönnerholm & Ann-Christine Syvänen

**DNA Methylation Assay**

Between 250 and 700 ng of genomic DNA was treated with sodium bisulfite according to the Illumina Infinium Assay protocol (EZ DNA methylation Gold, Zymo Research). The success of the bisulfite conversion was assessed using fully methylated and unmethylated control DNA samples that were bisulfite-converted in parallel. Methylated DNA from the human cell line HCT116 DKO (double knock-out) (Zymo Research) served as positive control and whole-genome amplified DNA served as negative control.

200 ng of bisulfite-converted DNA from each sample was subjected to analysis by the Infinium HumanMethylation 450k BeadChip assay (Illumina). An iScan SQ instrument (Illumina) was used for measuring the signals on the 450k BeadChips. The fluorescence intensities were extracted using the Methylation Module (1.8.5) software in Genome Studio (V2010.3) (Illumina). The two-color-fluorescence signals were normalized against the signals from >90 internal control probes. The average background signal derived from built-in negative control bead types was subtracted from the signals for each sample separately in the two fluorescence channels. The methylation levels (β-value) for each detectable C-residue were calculated by dividing the fluorescence signals corresponding to a methylated C-residue by the sum of the fluorescence signals corresponding to methylated and unmethylated C-residues. A β-value of zero indicates no methylation, and a β-value of 1.0 indicates complete methylation. A minimum detection p-value of 0.01 was implemented for all samples/probes, and probes that did not pass filter were masked. Paired diagnostic-remission or diagnostic-relapse samples were verified to originate from the same individual by genotyping 65 polymorphic SNP loci included on the 450k BeadChips. The β-value distribution between Infinium type I and II probes was normalized using peak-based correction (**Additional file 3, Figure S12**) [1].

**Probe Filtering**

Probes located on the X- and Y-chromosomes were removed from the downstream analysis to avoid sex-related methylation biases. The 65 SNP genotyping probes denoted with rs numbers included for DNA tracing were also removed. Probes with variable signals caused by unspecific genomic alignment or by genetic variants in the probe-binding sites that are affected by the probe hybridization reaction, and not variation in DNA methylation levels, should be excluded.

Firstly, we aligned the probe sequences of all autosomal probes to the human genome build 37 with BWA [2]. Probes mapping to multiple sites were indicated by a BWA mapping score <37 (n= 9,341). In 200 iterations, the SD in remission (non-leukemic) bone marrow aspirates from 86 pediatric ALL samples was calculated for 1,000 randomly selected probes that mapped to multiple sites and for 1,000 probes mapping to a single site. The difference in SD between the two probe sets was measured with a one sided Wilcoxon rank-sum test. Probes with non-unique alignments displayed significantly greater variance in β-values than uniquely mapping probes (**Additional file 3, Figure S13A**) and were excluded from further analysis.

Secondly, probes in the 450k DNA methylation assay overlapping single nucleotide polymorphisms (SNPs) and/or small insertion/deletions (indels) according to the dbSNP database (ver135) in their binding sites, which may affect the reported β-values were excluded. Using the same strategy as above, we compared the SD of probes in 86 non-leukemic samples with annotated SNPs to the SD in the same 86 non-leukemic samples for probes with no known underlying variation in a step-wise fashion, from the last 3’-position (CpG site in the case of Type II probes and the next base the case of Type I probes) up to the 10^th^ base from the 3’-end of the probe. The SD of probes with variants in the CpG site in the case of type II probes or the interrogation site in the case of type I probes, the 1^st^ base pair, and 2^nd^ base pair from the 3’ end of the probe displayed higher variability in β-values than probes without annotated SNPs (**Additional file 3, Figure S13B**) and were excluded from further analysis.

Based on these two analyses, probes mapping to multiple sites and probes with an annotated SNP within the probe binding region or interrogated base up to 2bp from the 3’ end of the probe were discarded from downstream analyses, resulting in a total of 435,941 autosomal CpG sites in the final data set.

**Technical Validation**

We benchmarked the performance of the Human Methylation 450k BeadChips by comparing the β-values obtained for 207 CpG sites to those determined by a custom-designed Golden Gate Methylation assay in 364 of the samples analyzed in the present study [3]. The mean coefficient of determination between the two assays across the 364 samples was R=0.92, indicating that the methylation values are highly reproducible between assays (**Additional file 3, Figure S14A-B**).

We also performed two technical and three biological replicate assays using the 450k BeadChips. By measuring the differences in DNA methylation values of the replicates, we estimated that differential methylation corresponding to a β-value ≥0.20 was detectable with >99.5% confidence (**Additional file 3, Figure S14C-H**).

**Digital Gene Expression (DGE) sequencing**

Data from previously published DGE sequencing libraries [4] from RNA from 23 of the diagnostic ALL samples included in the current study were analyzed together with newly generated DGE libraries from 11 additional diagnostic ALL patients and five non-leukemic control samples consisting of one CD19+ and four CD3+ cell samples as controls (**Additional file 2, Table S4**). The 11 additional DGE libraries were generated using the same methods as were previously described [4]. Briefly, 1 μg of high quality total RNA and reagents from the *NlaIII* Digital Gene Expression Tag Profiling kit (Illumina) were used. Polyadenylated RNA was captured on magnetic oligo(dT) beads and reverse into double stranded cDNA on the beads (SuperScript II, Invitrogen). The double stranded cDNA was cleaved using *NlaIII* and the first adapter was ligated to the double stranded cDNA fragments remaining on the beads after washing. The adapter contained the recognition sequence for Mme1, which after digestion released the cDNA from the magnetic bead, while leaving 17 bp of sequence in the fragment. After purification, the second adapter was ligated at the *MmeI* cleavage sites. Adapter-ligated cDNA fragments were amplified by PCR, and the products were gel purified. The ~96-bp PCR products were excised from the gel. Purified libraries were quality controlled and quantified on a Bioanalyzer using High-Sensitivity chips (Agilent).

Each DGE library was sequenced on an individual lane of a flow cell using an Illumina Genome Analyzer (GAII or GAIIx) for 18 cycles using reagents from version 2 cluster generation kits and version 3 sequencing kits (Illumina Inc.). Image analysis and base calling were performed using the Genome Analyzer pipeline v1.4. The first 17 bases of the tag sequences were extracted from the output files using a stringent base quality cutoff equivalent to a phred score of 20, discarding tags if they had any base with a score below 20.

DGE tags were annotated to the human transcriptome (Ensembl version 58) by mapping the reads to the sequence flanking *NlaIII* restriction sites on both coding and non-coding strands. Tags matching more than one gene were discarded. Tag counts were normalized to tags per million (TPM) by dividing the raw tag count by the total number of tags from each library and multiplying by one million. The total expression profile for each gene was calculated by summing all tags mapped to the same gene. Raw and normalized data are publicly available at the Gene Expression Omnibus (GEO) under accession number GSE26530 (<http://www.ncbi.nlm.nih.gov/geo/>).

**Array-based RNA quantification**

Two micrograms of total RNA from 93 of the 774 patient samples from ALL patient cells analyzed on the 450k DNA methylation array (**Additional file 2, Table S6**) was available for expression profiling on Human Genome U133 Plus 2.0 GeneChips (Affymetrix Inc., Santa Clara, CA, USA). Biotinylated cRNAs were hybridized to the gene chips for 16 hours in a 45°C incubator, rotated at 60 rpm. The GeneChips were then washed and stained using the Fluidics Station 450 and scanned using the GeneChip® Scanner 3000 7G (Affymetrix). The data was normalized using the robust multi-array average (RMA) method [5]. Raw and normalized data are publically available at GEO under series GSE47051. The genes were annotated using the HG-U133 Plus 2 Annotations release 33 (10/30/12). The normalized expression data for each probe-set was matched to the 450k data by gene symbols.

**Multivariate modeling for predicting relapse**

To identify patterns at diagnosis that are associated with future relapses the patients were divided into two classes: those without any event within 5 years from diagnosis and those who experienced a relapse within 5 years from diagnosis. Patients with shorter follow-up times and competing events within 5 years were not included in the modeling. Four sets of CpG-sites were used: constitutive DMCs (n=9,402), subtype-specific DMCs (n=271-16,841), subtype-specific DMCs correlated with gene expression (n=20-1,884), and relapse DMCs (n=6,612). The models based on the constitutive and relapse DMC signatures included all patients. In models based on subtype-specific DMCs and subtype-specific DMCs correlated with expression the patients were divided according to subtype. All analyses were further stratified based on patient treatment producing a total of 30 combinations of DMC signatures and patient groups analyzed separately (**Additional file 2, Table S17**). The modeling procedure consisted of three components: Model fitting and prediction, cross validation and permutation testing (**Additional file 3, Figure S8**).

The model fitting included a preprocessing step where CpG-sites with a difference in class-wise mean methylation <20% were discarded and missing values were imputed using kNN imputation with k selected as 5% of the number of samples (no test examples were included in the imputation). The test set had the same CpG-sites discarded as the design set and was imputed using the design set as reference. Nearest shrunken centroids classification models were fitted using the R-package “pamr” [6], with the modification that the shrinkage parameter was selected based on AUC (area under the ROC curve) due to unbalanced class sizes.

Repeated cross validation was used for robust estimation of the predictive performance of the classification and to extract the most informative CpG-sites. Performance was measured by AUC averaged across cross-validation folds. The CpG sites were scored by their coefficients, which are the shrunken standardized distance between the classes and the overall centroid, as defined in the pamr.listgenes function in the “pamr” package [6]. Prior to averaging, the scores in each fold were normalized to sum 1.0. This was implemented to reduce the large variability in magnitude of the scores due to different degrees of shrinkage across the folds.

The DMC signatures with mean AUC > 0.6 were tested for significance using permutation testing. The procedure described above was repeated a 1,000 times with randomly shuffled class memberships of the patients. The p-values were obtained by comparing the unpermuted mean AUC and mean CpG-site scores to the permuted equivalents,

$$p=\frac{n_{smaller}+1}{n+1}$$

where *n* is the number of permutations and *n_smaller_* is the number of permutations that produced an AUC or CpG-site score smaller than the unpermuted quantity (which is represented as the +1 terms).

**References**

1. Dedeurwaerder S, Defrance M, Calonne E, Denis H, Sotiriou C, Fuks F: **Evaluation of the Infinium Methylation 450K technology.** *Epigenomics* 2011, **3:**771-784.

2. Li H, Durbin R: **Fast and accurate short read alignment with Burrows-Wheeler transform.** *Bioinformatics* 2009, **25:**1754-1760.

3. Milani L, Lundmark A, Kiialainen A, Nordlund J, Flaegstad T, Forestier E, Heyman M, Jonmundsson G, Kanerva J, Schmiegelow K, et al: **DNA methylation for subtype classification and prediction of treatment outcome in patients with childhood acute lymphoblastic leukemia.** *Blood* 2010, **115:**1214-1225.

4. Nordlund J, Kiialainen A, Karlberg O, Berglund EC, Goransson-Kultima H, Sonderkaer M, Nielsen KL, Gustafsson MG, Behrendtz M, Forestier E, et al: **Digital gene expression profiling of primary acute lymphoblastic leukemia cells.** *Leukemia* 2012, **26:**1218-1227.

5. Irizarry RA, Hobbs B, Collin F, Beazer-Barclay YD, Antonellis KJ, Scherf U, Speed TP: **Exploration, normalization, and summaries of high density oligonucleotide array probe level data.** *Biostatistics* 2003, **4:**249-264.

6. Tibshirani R, Hastie T, Narasimhan B, Chu G: **Diagnosis of multiple cancer types by shrunken centroids of gene expression.** *Proc Natl Acad Sci U S A* 2002, **99:**6567-6572.
